# Phytochemical and pharmacological studies on *Solanum lyratum*: a review

**DOI:** 10.1007/s13659-022-00361-0

**Published:** 2022-11-09

**Authors:** Yue Zhao, Wen-Ke Gao, Xiang-Dong Wang, Li-Hua Zhang, Hai-Yang Yu, Hong-Hua Wu

**Affiliations:** State Key Laboratory of Component-Based Chinese Medicine, Institute of Traditional Chinese Medicine, Tianjin University of Traditional Chinese Medicine, 10 Poyanghu Road, West Area, Tuanbo New Town, Jinghai District, Tianjin, 301617 People’s Republic of China

**Keywords:** *Solanum lyratum* Thunb., Steroidal saponins, Steroidal alkaloids, Anti-cancer, Toxicity

## Abstract

*Solanum lyratum* is one of the temperate plants, broadly distributed in Korea, China, Japan, India, and South-East Asia and well-documented in those oriental ethnic medicine systems for curing cancers, jaundice, edema, gonorrhea, cholecystitis, phlogosis, rheumatoid arthritis, etc. This review systematically summarized the research progress on *S. lyratum* respecting the botany, traditional uses, phytochemistry, pharmacology, and toxicology to increase people's in-depth understanding of this plant, by data retrieval in a series of online or off-line electronic databases as far as we can reach. Steroidal saponins and alkaloids, terpenoids, nitrogenous compounds, and flavonoid compounds are the main chemical constituents in *S. lyratum*. Among them, steroidal alkaloids and saponins are the major active ingredients ever found in *S. lyratum*, exerting activities of anti-cancer, anti-inflammation, anti-microbial, anti-allergy, and anti-oxidation in vivo or in vitro. As a result, *S. lyratum* has been frequently prescribed for the abovementioned therapeutic purposes, and there are substantial traditional and modern shreds of evidence of its use.

**Graphical Abstract:**

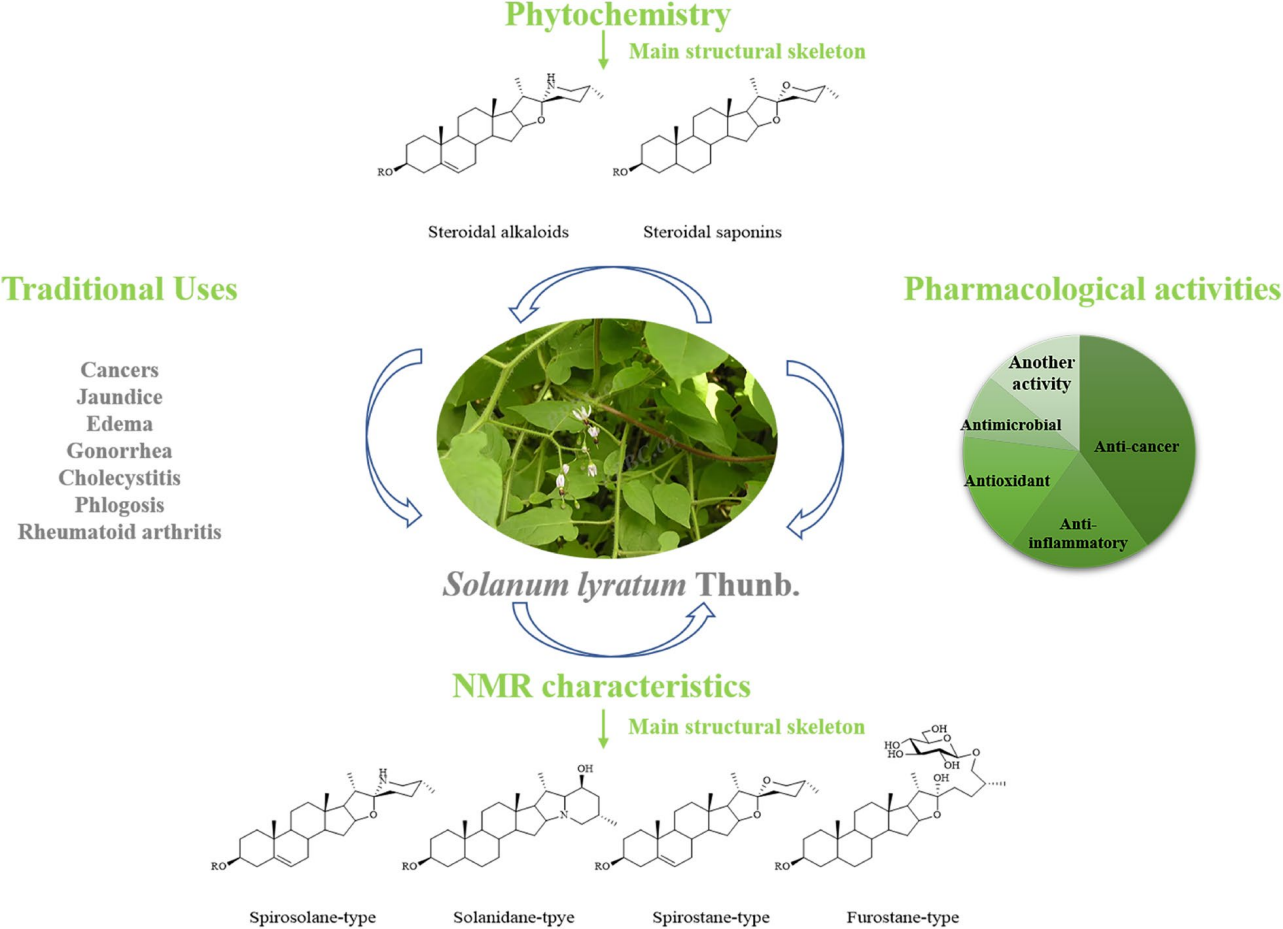

## Introduction

*Solanum lyratum* Thunb. is a herbaceous vine of the family Solanaceae, with white villous hairs on violin-shaped leaves and stems, distributed throughout China, Japan, Korea, etc. [1]. *S. lyratum* prefers a warm and humid environment and is distributed widely in valley grass, roadside and field. *S. lyratum* is commonly known as "Bai-Mao-Teng" in traditional Chinese medicine and "Back-Mo-Deung" in traditional Korean medicine [2]. In traditional Chinese medicine (TCM), *S. lyratum* has the functions of clearing heat and removing toxicity (“Qingre Jiedu” in Chinese), dispelling wind and eliminating dampness (“Qufeng Lishi” in Chinese). Therefore, *S. lyratum* has been traditionally prescribed mainly for healing jaundice, edema, gonorrhea, cholecystitis, phlogosis, and rheumatoid arthritis [3]. Modern phytochemistry and pharmacological studies revealed that *S. lyratum* consists of a variety of active ingredients, including steroidal saponins, steroidal alkaloids, terpenoids, lignans, and flavonoids [4]. And steroidal saponins and steroidal alkaloids have been used in the modern clinic to treat various cancers, especially lung cancer, cervical cancer, and liver cancer [3].

**Fig. 1:**
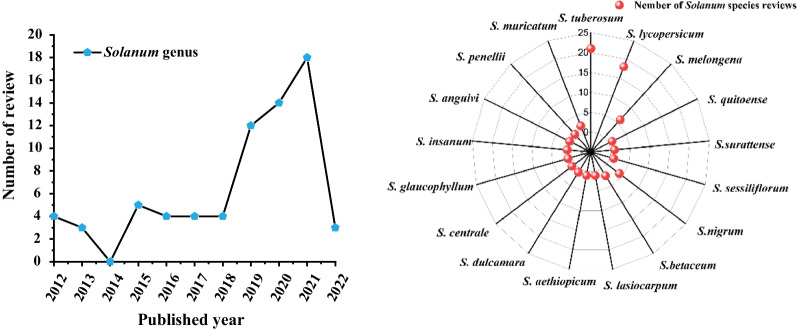
Reviews of *Solanum* species published in last ten years

In the last ten years, dozens of reviews on the research progress of *Solanum* plants have been published, occasionally referring few phytochemistry and pharmacological reports on *S. lyratum* [5–8] (Fig. 1). However, there is no specialized and systematic research review on the *S. lyratum* species, especially on its phytochemistry and pharmacological aspects. Thus, this review intends to provide an updated and comprehensive summary on the botanical characterization, phytochemistry, and pharmacological and toxicity studies of *S. lyratum* to fill a gap in the research review of this plant and provides for a better exploration and application of *S. lyratum*. The literature for this manuscript was obtained from reports published from 1981 to Mar 2022.

## Botany

*S. lyratum* is a herbaceous vine of the family Solanaceae, well-known native in China, India, Japan, Korea, North Vietnam, and the Indochina Peninsula [1]. This plant grows in a warm and humid environment, prefers light and fertile organic soil, and is distributed on hillsides, grass, ditch, and roadside at altitudes of 100–850 m. *S. lyratum* is 0.5–1 m long, and its stems and twigs are densely covered with white villous hairs [2]. The botanical characteristics of *S. lyratum* were recorded in many classics of TCM, including "*Tang Xinxiu Bencao*" in the Tang Dynasty [9], "*Zhenglei Bencao*" in the Song Dynasty [10], and "*Compendium of Materia Medica*" in Ming Dynasty [11].

**Fig. 2:**
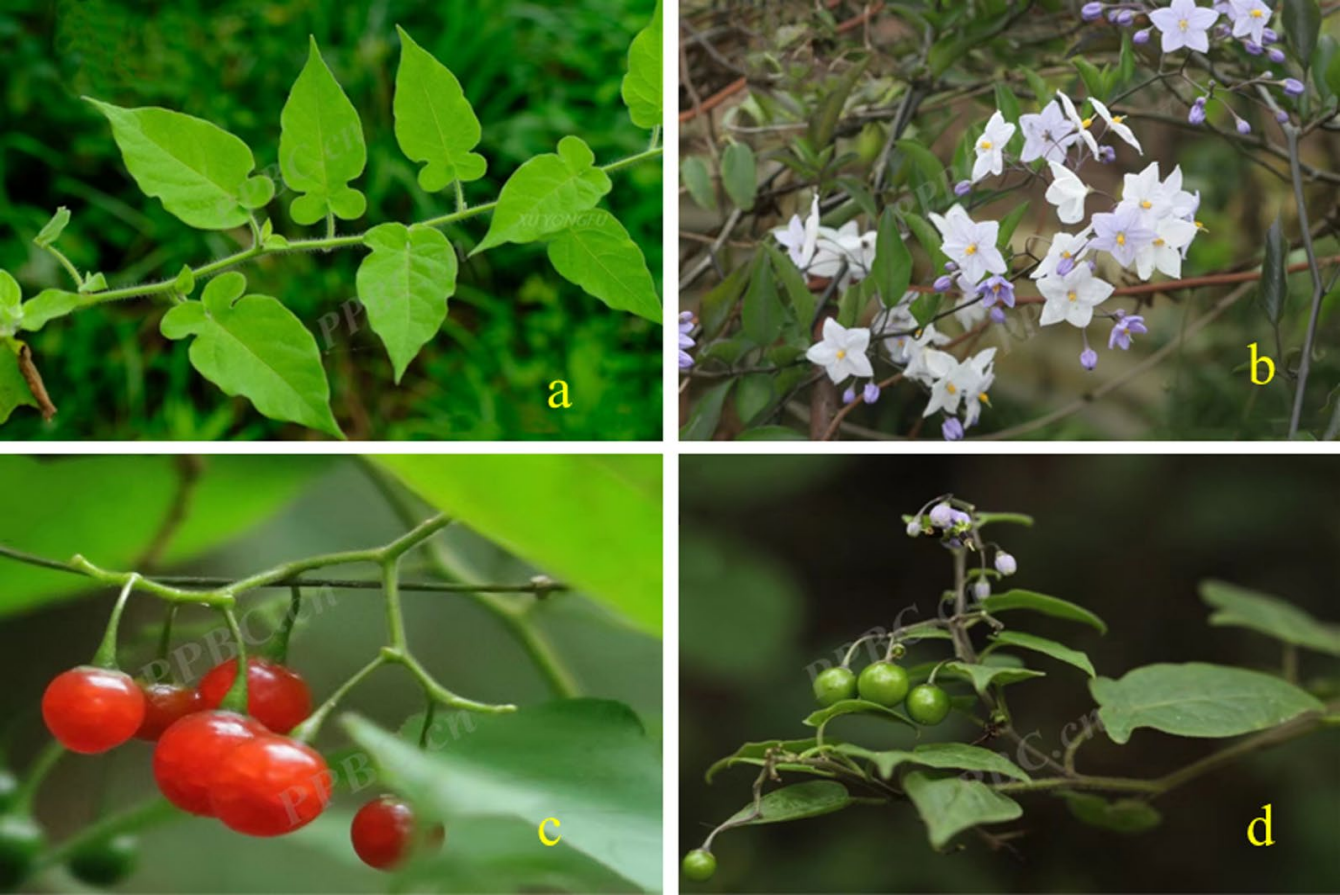
Plant morphology of *S. lyratum*, (a) leaves, (b) flowers (c) fruits (d) the whole plant [data originated from the Plant Photo Bank of China (http://ppbc.iplant.cn/), accessed 10 April 2022]

*S. lyratum* is commonly known as "Bai-Mao-Teng" in TCM. It should be noted that there are several adulterants of *S. lyratum*, including *Aristolochia mollissima*, *Paederia scandens* (Lour.) Merr. and *Solanumrr dulcamara* L., all of which are so called as "Bai-Mao-Teng" that it may easily cause an event of medication confusion [12]. For example, a misuse of *A. mollissima* instead of *S. lyratum*, has ever led to a renal failure event in patients of Hong Kong [13]. Those adulterants closely resemble *S. lyratum* in botanical morphology, it is very important to seek advice from a professional or pharmacist before use. The plant morphological characteristics of *S. lyratum* are as follows: Root is slender and cylindrical. Leaves are mostly violin-shaped, with 3.5–5.5 cm long and 2.5–4.8 cm wide, and the base is 3–5 cm deep-lobed. Lateral lobes are smaller near the base. Middle lobes are usually larger oval and tend to apex acuminate. Both sides of the leaves were covered with white shiny villous hairs, and the levels own midvein and lateral veins. Flowers are sparsely terminal inflorescence or extra-axillary inflorescence, and the pedicel is approximately 2–2.5 cm long. Corollas are blue-purple or white and corollas are about 1.1 cm in diameter. Fruits are spherical and about 8 cm in diameter, which become reddish-black when it matures. Seeds are nearly disc-shaped and about 1.5 mm in diameter. The flowering period of *S. lyratum* is between May and June, while the fruiting period is between August and October. Significantly, the suitable harvest time has been recommended to be between October and December (Fig. 2) [14, 17].

## Traditional uses

In TCM, *S. lyratum* has been considered as one of the "Top-grade" herbs in "*Shennong Bencao Jing*" (100 BC-200 AD, Han Dynasties) [18]. For centuries, it has been used for the treatments of cold and fever, malaria, jaundice, nephritis, edema, cholecystitis, rheumatoid arthritis, vaginitis, uterine erosion, and several types of cancer including lung cancer, cervical cancer, and gastric cancer [19, 21]. External applications of *S. lyratum* [22] have been recorded to treat carbuncle, furuncle and swollen poison, etc. In the "*Compendium of Materia Medica*", *S. lyratum* is documented [11] to have the effects of clearing heat, detoxification, and expelling rheumatism, for the treatment of rubella, erysipelas, malaria, cancer, etc. The traditional uses of *S. lyratum* in Korea, Japan, and the Indochina Peninsula focused mainly on the treatments of several types of cancers, warts, herpes, pyretic syndrome, diarrhea, etc*.*, as summarized in Table 1.

**Table 1:** Traditional uses of *S. lyratum*

Plant part used	Place	Traditional uses	Refs.
Herba, fruit	People's Republic of China	Clearing heat and removing toxicity, dispelling wind, malaria, jaundice, nephritis, edema, cholecystitis, rheumatoid arthritis, vaginitis, uterine erosion, carbuncle, cancer, diarrhea, wind-fire toothache, vaginitis	[17, 19–21]
Herba	Europe	Cancers, tumors, herpes and warts	[23, 24]
Herba	Korea	Febrifuge, diarrhea, eye disease, cancer, antitumor, anti-inflammatory, immunomodulatory, anti-anaphylactic, antioxidant agent, pyretic syndrome, diarrhea	[19, 21, 25–27]
Herba	Japan	Cancer, herpes, clearing heat and detoxifying, dispelling wind and resolving phlegm, eliminating dampness and removing jaundice, diarrhea, eye disease, pyretic syndrome	[19, 21, 27, 28]
Herba, Leaf	Taiwan, People's Republic of China	Leukemia, liver cancer, lung cancer, esophagus cancer, tumors, and warts	[29, 30]
Herba	Indochina Peninsula	Cancer, tumor, rheumatoid arthritis, leucorrhea, cold fever, damp-heat jaundice, herpes, and nephritis dropsy, pyretic syndrome, diarrhea	[21, 31]

## Phytochemistry

So far, hundreds of phytochemicals have been isolated and identified from *S. lyratum*, including steroidal alkaloids (**1**–**41**), steroids and steroidal saponins (**42**–**101**), terpenoids (**102**–**153**), nitrogenous compounds (**154**–**178**), phenylpropanoids (**179**–**227**), flavonoids (**228**–**258**) and other compounds (**259**–**270**). Among them, steroidal alkaloids, steroidal saponins and terpenoids are so often recognized as the main active constituents of *S. lyratum* [32, 33].

### Steroidal alkaloids

**Fig. 3:**
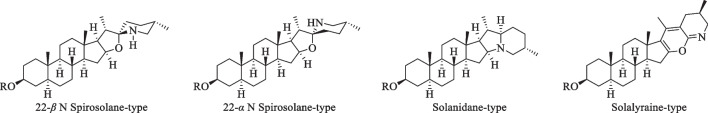
Three types of steroidal alkaloid aglycones reported in *S. lyratum*

**Fig. 4:**
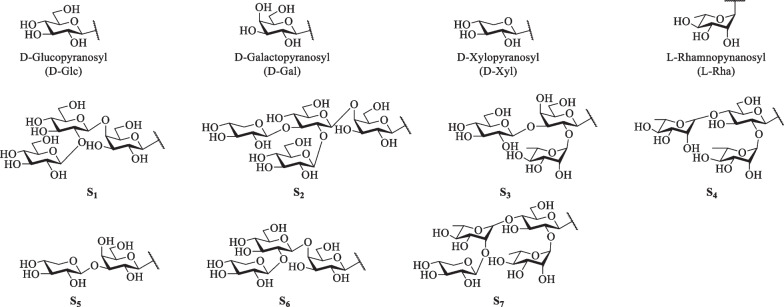
Glycones of steroidal alkaloids reported in *S. lyratum*

Steroidal alkaloids in *S. lyratum* include mostly solanidane (27 carbon atoms), spirosolane (27 carbon atoms) and solayraine (27 carbon atoms) (Fig. 3) types of nitrogenous sapogenins. The glycone moieties are most likely to be substituted at C-3 position of the nitrogenous sapogenin aglycone. D-glucose (D-Glc), D-galactose (D-Gal), D-xylose (D-Xyl), and L-rhamnose (L-Rha) are the common components of the glycones, in which one to four monosaccharides linked linearly or with one or more branched chains, as shown in Fig. 4.

**Fig. 5:**
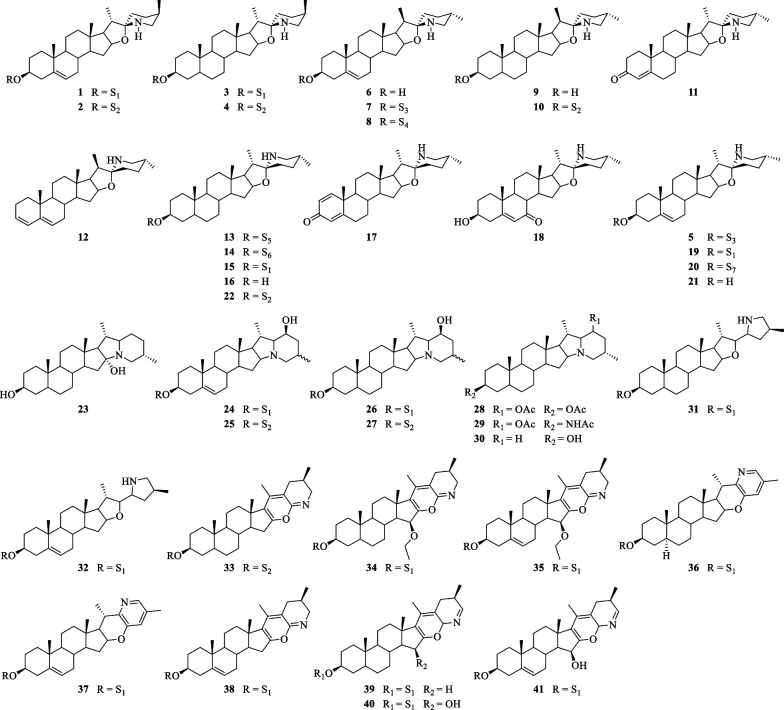
Chemical structures of steroidal alkaloids from *S. lyratum*

Steroidal alkaloid is one of the characteristic ingredients of *Solanum* plants [34]. Until now, a total of forty-one steroidal alkaloids (**1**–**41**) have been identified from *S. lyratum* (Table 2). It is noteworthy that there were two epimers for the most abundant spirosolane-type steroidal alkaloids in *Solanum* plants, one is 22-*β N* type (**1**–**4**, **6**–**11**) [33, 35–39] and the other is 22-*α N* type (**5**, **13**–**22**) [24, 35–40] in clue of the existence of an oxa-azaspirodecane system. In addition, solanidane-type steroidal alkaloids (**23**–**30**) [35, 36, 41, 42], with a unique octahydroindolizine complex cholestane skeleton, have also been found to be existed in this plant. Further, other unusual spirosolane-type glycoalkaloids with a deformed E and F rings (piperidine, pyridine or other derived F rings) have been also occasionally discovered from *S. lyratum*, exemplified by compounds **31**–**41** [39, 43], as shown in Fig. 5.

**Table 2:** Steroidal alkaloids isolated from *S. lyratum*

**No**	**Compounds**	**Chemical formula**	**Molecular Wt**	**Refs.**
**1**	(3*β*,22*α*,25*R*)-Spirosol-5-en-3-ol-*O*-*β*-D-glucopyranosyl-(1 → 2)-*O*-*β*-D-glucopyranosyl-(1 → 4)-*O*-*β*-D-galactopyranoside	C_45_H_73_NO_17_	899.4878	[33]
**2**	(3*β*,22*α*,25*R*)-Spirosol-5-en-3-ol-*O*-*β*-D-glucopyranosyl-(1 → 2)-[*O*-*β*-D-xylopyranosyl-(1 → 3)]-*O*-*β*-D-glucopyranosyl-(1 → 4)-*O*-*β*-D-galactopyranoside	C_50_H_81_NO_21_	1031.5301	[33]
**3**	(3*β*,5*α*,22*α*,25*R*)-Spirosol-3-ol-*O*-*β*-D-glucopyranosyl-(1 → 2)-*O*-*β*-D-glucopyranosyl-(1 → 4)-*O*-*β*-D-galactopyranoside	C_45_H_75_NO_17_	901.5035	[33]
**4**	(3*β*,5*α*,22*α*,25*R*)-Spirosol-3-ol-*O*-*β*-D-glucopyranosyl-(1 → 2)-[*O*-*β*-D-xylopyranosyl-(1 → 3)]-*O*-*β*-D-glucopyranosyl-(1 → 4)-*O*-*β*-D-galactopyranoside	C_50_H_83_NO_21_	1033.5458	[33]
**5**	Solasonine	C_45_H_75_NO_16_	885.5086	[35]
**6**	Tomatidenol	C_27_H_43_NO_2_	413.3294	[36]
**7**	Solamarine	C_45_H_73_NO_16_	883.4929	[35]
**8**	Solamargine	C_45_H_73_NO_15_	867.4980	[35, 37]
**9**	Soladulcidine	C_27_H_45_NO_2_	415.3450	[35, 37]
**10**	Soladulcidine-3-*O*-*β*-D-glucopyranosyl-(1 → 2)-[*O*-*β*-D-xylopyranosyl-(1 → 3)]-*O*-*β*-D-glucopyranosyl-(1 → 4)-*O*-*β*-D-galactopyranoside	C_50_H_83_NO_21_	1033.5458	[34, 38]
**11**	4-Tomatiden-3-one	C_27_H_41_NO_2_	411.3137	[36]
**12**	Solasodiene	C_27_H_41_NO	395.3188	[35]
**13**	Solalyratine A	C_38_H_62_NO_11_	709.4401	[24]
**14**	Solalyratine B	C_44_H_73_NO_16_	871.4929	[24]
**15**	Solalyratine B’	C_45_H_75_NO_17_	901.5035	[39]
**16**	Soladulcidine	C_27_H_45_NO_2_	415.3450	[40]
**17**	1,4-Solasodadien-3-one	C_27_H_39_NO_2_	409.2981	[36]
**18**	7-Oxosolasodine	C_27_H_41_NO_3_	427.3086	[36]
**19**	Solalyraine A’	C_45_H_73_NO_17_	899.4878	[39]
**20**	(3*β*,22*β*,25*S*)-Spirosol-5-ene-3-*O*-*β*-D-xylopyranosyl-(1 → 2)-*O*-*α*-L-rhamnopynanosyl-(1 → 4)-[*O*-*α*-L-rhamnopynanosyl-(1 → 2)]-*O*-*β*-D-glucopyranoside	C_50_H_81_NO_19_	999.5403	[35]
**21**	Solasodine	C_27_H_43_NO_2_	413.3294	[36]
**22**	Solalyratine C	C_50_H_83_NO_21_	1033.5458	[35]
**23**	5*α*-Solanidane-3*β*,16*α*-diol	C_27_H_45_NO_2_	415.3450	[36]
**24**	(25*S* or *R*)-Solanid-5-ene-3*β*.23*β*-diol-3-*O*-*β*-D-glucopyranosyl-(1 → 2)-*O*-*β*-D-glucopyranosyl-(1 → 4)-*O*-*β*-D-galactopyranoside	C_45_H_73_NO_17_	899.4878	[41]
**25**	(25*S* or *R*)-Solanid-5-ene-3*β*,23*β*-diol-3-*O*-*β*-D- glucopyranosyl-(1 → 2)-[xylopyranosyl-(1,3)]-*O*-*β*-D-glucopyranosyl-(1 → 4)-*O*-*β*-D-galactopyranoside	C_50_H_81_NO_21_	1031.5301	[35]
**26**	(25*S* or *R*)-Solanidane-3*β*.23*β*-diol-3-*O*-*β*-D-glucopyranosyl-(1 → 2)-*O*-*β*-D-glucopyranosyl-(1 → 4)-*O*-*β*-D-galactopyranoside	C_45_H_75_NO_17_	901.5035	[41]
**27**	(25*S* or *R*)-Solanidane-3*β*.23*β*-diol-3-*O*-*β*-D- glucopyranosyl-(1 → 2)-[xylopyranosyl-(1 → 3)]-*O*-*β*-D-glucopyranosyl-(1 → 4)-*O*-*β*-D-galactopyranoside	C_50_H_83_NO_21_	1033.5458	[35, 41]
**28**	Dihydroleptinidin diacetate	C_31_H_49_NO_4_	499.3662	[41]
**29**	Solanogantamine diacetate	C_31_H_50_N_2_O_3_	498.3821	[41]
**30**	Dihydroleptinidine	C_27_H_45_NO	399.3501	[42]
**31**	Solalyraine A	C_45_H_75_NO_17_	901.5035	[43]
**32**	Solalyraine B	C_45_H_73_NO_17_	899.4878	[43]
**33**	(3*β*,5*α*,25*S*)-16,23-Epoxy-23,24-iminocholest-16,20,23(*N*)-triene-3-*O*-*β*-D-glucopyranosyl-(1 → 2)-[*β*-D-xylopyranosyl-(1 → 3)]-*O*-*β*-D-glucopyranosyl-(1 → 4)-*O*-*β*-D-galactopyranoside	C_50_H_77_NO_21_	1027.4988	[39]
**34**	15*β*-Hydroxyl-(3*β*,25*R*)-16,23-epoxyl-23,24-iminocholestane-5,16,20,23(*N*)-tetraen-3*β*-ol-3-*O*-*β*-D-glucopyranosyl-(1 → 2)-*O*-*β*-D-glucopyranosyl-(1 → 4)-*O*-*β*-D-galactopyranoside	C_47_H_74_NO_18_	940.4931	[39]
**35**	15*β*-Ethoxyl-(3*β*,5*α*,25*R*)-16,23-epoxyl-23,24-iminocholest-16,20,23(*N*)-trien-3*β*-ol-3-*O*-*β*-D-glucopyranosyl-(1 → 2)-*O*-*β*-D-glucopyranosyl-(1 → 4)-*O*-*β*-D-galactopyranoside	C_47_H_72_NO_18_	938.4790	[39]
**36**	16,23-Epoxyl-22,26-iminocholestane-22(*N*),23,25(26)-trien-3*β*-ol-3-*O*-*β*-D-glucopyranosyl-(1 → 2)-*O*-*β*-D-glucopyranosyl-(1 → 4)-*O*-*β*-D-galactopyranoside	C_45_H_69_NO_17_	895.4565	[39]
**37**	Solalyraine C	C_45_H_67_NO_17_	893.4409	[43]
**38**	Solalyraine D	C_45_H_67_NO_17_	893.4409	[43]
**39**	Solalyraine E	C_45_H_69_NO_17_	895.4565	[43]
**40**	Solalyraine F	C_45_H_69_NO_18_	911.4515	[43]
**41**	Solalyraine G	C_45_H_67_NO_18_	909.4358	[43]

### NMR characteristics of steroidal alkaloids

Representative NMR data of the common steroidal alkaloids and saponins from *S. lyratum* were summarized in Tables 3, 4, 5 and 6. ^13^C NMR spectra of the spirosolane-type, spirostanol-type, and furostanol-type steroidal alkaloids or saponins, with intrinsic twenty-seven steroid skeleton, normally exhibit characteristic carbon signals for C-22 around *δ*_C_ 98.0 [35], 109.0 [22], and 112.0 [40] ppm, respectively. In addition, in the high field of the ^1^H NMR spectra of steroidal alkaloids and saponins, the resonance signals of methylene and methine protons are generally around *δ*_H_ 1.1–3.0, while the four methyl groups show proton resonances at *δ*_H_ 0.6–1.4, among which there are two singlets for the methyl groups at C-18 and C-19 [44], and two doublets for those methyls at C-21 and C-27 [45].

**Table 3:** Representative ^13^C NMR data of six common steroid alkaloids

Position	Compound **8**^*a*^ (spirosolane-type)	Compound **4**^*b*^ (spirosolane-type)	Compound **19**^b^ (spirosolane-type)	Compound **26**^*c*^ (solanidane-type)	Compound **24**^*c*^ (solanidane-type)	Compound **33**^*b*^ (solalyraine-type)
**1**	37.6	38.2	38.5	37.1	37.3	29.7
**2**	30.3	30.4	30.7	31.5	31.5	30.4
**3**	78.1	79.3	79.9	71.2	71.6	79.3
**4**	39.0	35.3	39.6	38.2	42.3	30.4
**5**	148.0	46.0	142.1	45.0	141.0	46.1
**6**	121.9	29.8	122.3	28.7	121.3	32.9
**7**	32.7	33.2	33.1	32.3	32.1	35.3
**8**	31.8	36.5	32.8	35.4	31.7	38.0
**9**	50.4	55.6	51.5	54.5	50.2	55.7
**10**	37.2	36.8	38.0	35.6	36.6	36.9
**11**	21.3	22.1	21.9	21.0	20.8	22.2
**12**	40.2	40.6	40.4	39.6	39.4	37.1
**13**	40.7	42.4	42.2	41.4	41.4	48.1
**14**	56.8	57.5	57.7	57.4	57.7	57.2
**15**	32.5	33.0	33.0	31.5	31.5	35.2
**16**	78.9	84.7	84.8	69.6	69.6	138.7
**17**	63.6	63.2	63.0	62.2	62.2	149.8
**18**	16.7	16.7	16.5	16.8	16.6	17.1
**19**	19.5	12.7	19.8	12.4	19.4	12.6
**20**	41.7	42.8	42.8	30.6	30.6	141.4
**21**	15.8	14.7	14.9	18.9	18.9	12.3
**22**	98.5	100.2	100.2	78.9	78.9	162.6
**23**	34.8	33.4	33.2	67.0	67.0	141.7
**24**	31.2	28.9	28.9	37.1	37.1	63.2
**25**	31.7	29.5	29.5	26.9	26.9	31.6
**26**	48.2	46.7	46.7	58.7	58.7	38.2
**27**	19.9	18.6	18.6	22.4	22.4	16.6
Gal-(1 → 3)-skeleton						
**1'**		102.7	102.8	102.3	102.3	102.7
**2'**		73.2	73.2	73.1	73.1	73.2
**3'**		75.3	75.2	75.4	75.4	75.3
**4'**		80.2	80.5	80.8	80.8	80.2
**5'**		75.9	76.3	76.5	76.5	75.9
**6'**		61.1	60.9	60.4	60.4	61.1
Glc-(1 → 3)-skeleton						
**1'**	100.3					
**2'**	78.0					
**3'**	77.8					
**4'**	78.6					
**5'**	77.0					
**6'**	61.3					
Rha'-(1 → 4)-Gal						
**1''**	102.1					
**2''**	72.6					
**3''**	72.9					
**4''**	74.2					
**5''**	69.6					
**6''**	18.8					
Rha''-(1 → 2)-Gal						
**1''**	102.9					
**2''**	72.6					
**3''**	72.8					
**4''**	74.0					
**5''**	70.5					
**6''**	18.6					
Glc'-(1 → 4)-Gal						
**1''**		104.3	104.9	105.0	105.0	104.3
**2''**		81.0	85.0	85.8	85.8	81.1
**3''**		87.9	77.6	78.0	78.0	87.9
**4''**		71.0	70.7	71.7	71.7	71.0
**5''**		78.3	78.2	77.4	77.4	78.3
**6''**		62.7	62.0	61.4	61.4	62.0
Glc''-(1 → 2)-Glc'						
**1'''**		104.7	106.2	106.7	106.7	104.7
**2'''**		75.6	75.6	74.9	74.9	75.6
**3'''**		78.5	78.7	78.2	78.2	78.5
**4'''**		71.6	71.8	70.2	70.2	71.6
**5'''**		78.0	77.9	77.4	77.4	78.0
**6'''**		63.2	63.2	63.1	63.1	63.1
Xyl-(1 → 3)-Glc'						
**1'''**		105.0			104.8	105.0
**2'''**		75.2			75.5	75.3
**3'''**		77.5			78.5	77.5
**4'''**		70.4			70.4	70.5
**5'''**		67.2			67.2	67.2

^*a*^In C_5_D_5_N; ^*b*^In CD_3_OD; ^*c*^In CDCl_3_

**Table 4:** Steroids isolated from *S. lyratum*

No	Compounds	Chemical formula	Molecular Wt	Refs.
42	Tigogenin	C_27_H_44_O_3_	416.3290	[47]
43	Diosgenin	C_27_H_42_O_3_	414.3134	[47]
44	(25*R*)-Spirost-4-ene-3,12-dione	C_27_H_38_O_4_	426.2770	[47]
45	(25*R*)-Spirostane-4,6-dien-3-one	C_27_H_38_O_3_	410.2821	[47]
46	(25*R*)-Spirost-4-en-3-one	C_27_H_40_O_3_	412.1977	[47]
47	7-Ketodiosgenin	C_27_H_40_O_4_	428.2927	[47]
48	Agigenin	C_27_H_44_O_5_	448.3189	[47]
49	Hecogenin	C_27_H_42_O_4_	430.3083	[47]
50	***△***^5(6)^-22-Isospirostene-2,3-diol	C_27_H_42_O_4_	430.3038	[47]
51	Gitogenin	C_27_H_44_O_4_	432.3240	[47]
52	20-Hydroxydiosgenone	C_27_H_40_O_4_	428.2927	[47]
53	(25*R*)-25-Hydroxyspirost-4-en-3-one	C_27_H_42_O_4_	430.3083	[47]
54	Tigogenone	C_27_H_42_O_3_	414.3134	[50]
55	***△***^3,5^-deoxytigogenin-(25*R*)-spirost-3,5-diene	C_27_H_40_O_2_	396.3208	[48]
56	Diosgenin	C_27_H_42_O_2_	398.3185	[35]
57	Yamogenin	C_27_H_42_O_2_	398.3185	[35]
58	Periplogenin	C_23_H_34_O_5_	390.2406	[47]
59	16-Dehydropregnenolone	C_21_H_30_O_2_	314.2246	[51]
60	3-Hydroxy-5-pregn-16-en-20-one	C_21_H_32_O_2_	316.2402	[51]
61	3*β*,6*α*,16*β*-Trihydroxy-5*α*-pregnane-(20*S*)-carboxylic acid (22,16)-lactone	C_22_H_32_O_3_	344.2351	[47]
62	24-Methylcholest-5-en-3,16-diol	C_28_H_48_O_2_	416.3654	[47]
63	Cholesterol	C_27_H_46_O	386.3549	[40]
64	5*α*-Stigmanstane-3,6-dione	C_29_H_48_O_2_	428.3654	[40]
65	4-Methylcholest-7-en-3*β*-ol	C_28_H_48_O	400.3705	[50]
66	24*α*-Methylcholestane-7,22-diene-3*β*,5*α*,6*β*-triol	C_28_H_46_O_3_	430.3447	[36]
67	5*α*-Stigmanstane-3-hydroxy-6-dione	C_29_H_50_O_2_	430.3811	[40]
68	*β*-Sitosterol	C_30_H_52_O	428.7330	[44]
69	Daucosterol	C_35_H_60_O_6_	576.4390	[44]
70	Ergosterol endoperoxide	C_28_H_44_O_3_	428.3290	[52]
71	9,11-Dehydroergosterol endoperoxide	C_28_H_42_O_3_	426.3134	[52]

**Table 5:** Steroidal saponins isolated from *S. lyratum*

No	Compounds	Chemical formula	Molecular Wt	Refs.
**72**	Diosgenin-3-*O*-*β*-D-glucopyranosyl-(1 → 3)-[*O*-*β*-D-glucopyranosyl-(1 → 2)]-*O*-*β*-D-glucopyranosyl-(1 → 4)-*O*-*β*-D-galcopyranoside	C_51_H_84_O_23_	1064.5403	[22]
**73**	Diosgenin-3-*O*-*β*-D-xylopyranosyl-(1 → 3)-[*O*-*β*-D-glucopyranosyl-(1 → 2)]-*O*-*β*-D-glucopyranosyl-(1 → 4)-*O*-*β*-D-glucopyranoside	C_50_H_82_O_22_	1034.5298	[22]
**74**	Diosgenin-3-*O*-*β*-D-glucopyranosiduronic acid methyl ester	C_34_H_52_O_9_	604.3611	[47]
**75**	Diosgenin-3-*O*-*α*-L-rhamnosyl-(1 → 2)-*O-β*-D-glucopyranosiduronic acid	C_39_H_60_O_13_	736.4034	[47]
**76**	Diosgenin-3-*O*-*α*-L-rhamnosyl-(1 → 2)-*O*-*β*-D-glucopyranosiduronic acid methyl ester	C_40_H_62_O_13_	750.1490	[47]
**77**	Diosgenin-3-*O*-*β*-D-glucopyranosiduronic acid	C_33_H_50_O_9_	590.3455	[47]
**78**	Diosgenin-3-*O*-*β*-D-xylopyranosyl-(1 → 3)-*O*-*β*-D-glucopyranosy1-(1 → 4)-[*O*-*α*-L-rhamnopyranosyl-(1 → 2)]-*O*-*β*-D-glucopyranoside	C_50_H_80_O_21_	1016.5192	[47]
**79**	Diosgenin-3-*O*-*β*-D-glucopyranosyl-(1 → 4)-*O*-*β*-D-galactopyranoside	C_39_H_62_O_13_	738.4190	[47, 49]
**80**	Diosgenin-3-*O*-*β*-D-glucopyranosy1-(1 → 3)-[*O*-*α* -L-rhamnopyranosyl-(1 → 2)]-*O*-*β*-D-glucopyranoside	C_45_H_72_O_17_	884.4770	[4]
**81**	(25*R*)-Spirost-5-en-3*β*-ol-*O*-*β*-D-glucopyranosyl-(1 → 4)-[*O*-*α*-L-rhmanopyranosyl-(1 → 2)]-*O*-*β*-D-galactopyranoside	C_45_H_72_O_17_	884.4770	[22]
**82**	Funkioside D	C_45_H_72_O_18_	900.4719	[22]
**83**	Aspidistrin	C_51_H_84_O_22_	1048.5454	[22]
**84**	(25*R*)-Spirost-5-en-3*β*-ol-*O*-*β*-D-glucopyranosyl-(1 → 3)-[*O*-*α*-L-rhmanopyranosyl-(1 → 2)]-*O*-*β*-D-glucopyranosiduronic acid methyl ester	C_46_H_72_O_18_	912.4719	[4]
**85**	Gitogenin-3-*O*-*β*-D-glucopyranosyl-(1 → 4)-*O*-*β*-D-galactopyranoside	C_39_H_64_O_13_	740.4347	[47]
**86**	(3*β*,25*S*)-Spirost-5-ene-3-*O*-*β*-D-glucopyranosyl-(1 → 2)-*O*-*β*-D-glucopyranosyl-(1 → 4)-*O*-*β*-D-galactopyranoside	C_45_H_74_O_18_	902.4875	[46]
**87**	(3*β*,25*S*)-Spirit-5-ene-3-*O*-*β*-D-glucopyranosyl-(1 → 3)-*O*-*β*-D-glucopyranosyl-(1 → 4)-*O*-*β*-D-galactopyranoside	C_45_H_72_O_18_	900.4719	[47]
**88**	Lyratoside D	C_33_H_52_O_9_	592.3611	[40]
**89**	16-Dehydropregnenolone-3-*O*-*α*-L-rhamnopyranosyl-(1 → 2)-*O*-*β*-D-glucopyranosiduronic acid	C_33_H_48_O_12_	636.3146	[51]
**90**	Lyratoside E	C_39_H_60_O_16_	784.3881	[40]
**91**	Lyratoside F	C_39_H_60_O_17_	800.3831	[40]
**92**	5*α*-Pregn-16-en-3*β*-ol-20-one-3-*O*-*β*-D-glucopyranosyl-(1 → 2)-*O*-*β*-D-glucopyranosyl-(1 → 4)-*O*-*β*-D-galactopyranoside	C_39_H_62_O_17_	802.3987	[46]
**93**	Pallyidifloside B	C_45_H_72_O_19_	916.4668	[40]
**94**	26-*O*-*β*-D-Glucopyranosyl-(22*S*,25*S*)-3*β*,26-dihydroxy-22-methoxyfurost-5-ene-3-*O*-*α*-L-rhamnose-(1 → 2)-3-*O*-*β*-D-glucuronopyranoside	C_46_H_74_O_19_	930.4824	[23]
**95**	26-*O*-*β*-D-Glucopyranosyl-(22*S*,25*S*)-3*β*,26-dihydroxy-22-methoxyfurost-5-ene-3-*O*-*β*-D-glucopyranosyl-(1 → 3)-[*O*-*α*-L-rhamnose-(1 → 2)]-3-*O*-*β*-D-glucuronopyranoside	C_51_H_82_O_24_	1078.5196	[23]
**96**	26-*O*-*β*-D-Glucopyranosyl-(22,25*R*)-3*β*,26-dihydroxy-22-methoxyfurost-5-ene-3-*O*-*α*-L-rhamnopyranosyl-(1 → 2)-*O*-*β*-D-glucopyranosiduronic acid methyl ester	C_47_H_76_O_19_	944.5247	[40]
**97**	Lyratoside C	C_52_H_86_O_24_	1094.5509	[40]
**98**	26-*O*-*β*-D-Glucopyranosylfurostane-3,22,26-triol-3-*O*-*β*-D-glucopyranosyl-(1→2)-*O*-*β*-D-glucopyranosyl-(1→4)-*O*-*β*-D-galactopyranoside	C_51_H_86_O_24_	1082.5509	[46]
**99**	26-*O*-*β*-D-Glucopyranosyl-22-methoxyfurost-3,26-diol-3-*O*-*β*-D-glucopyranosyl-(1→2)-*O*-*β*-D-glucopyranosyl-(1→4)-*O*-*β*-D-galactopyranoside	C_52_H_88_O_24_	1096.5666	[46]
**100**	26-*O*-*β*-D-Glucopyranosyl-(25*R*)-5,20(22)-dienefurostane-3*β*,26-diol	C_33_H_52_O_8_	576.3662	[22]
**101**	26-*O*-*β*-D-Glucopyranosyl-(25*R*)-5*α*-furost-20(22)-ene-3*β*,26-diol	C_33_H_54_O_8_	578.3819	[22]

**Table 6:** Representative ^13^C NMR data of six common steroidal saponins (in C_5_H_5_N-*d*_*5*_)

**Position**	Compound **73** (spirostanol-type)	Compound **79 **(spirostanol-type)	Compound **60** (C_21_-steroid-type)	Compound **91** (C_21_-steroid-type)	Compound **97** (furostanol-type)	Compound **99** (furostanol-type)
**1**	37.2	37.6	32.5	37.3	37.4	37.2
**2**	29.9	30.4	32.2	30.2	30.2	30.8
**3**	78.5	78.4	70.6	78.0	77.2	77.4
**4**	34.8	39.4	37.3	39.3	39.2	34.8
**5**	44.7	141.2	45.5	141.4	141.0	44.7
**6**	28.9	121.6	29.1	121.3	121.6	28.9
**7**	32.4	32.4	32.3	31.7	32.1	32.4
**8**	35.3	31.9	34.0	30.3	31.6	35.2
**9**	54.4	50.5	56.6	50.7	50.2	54.4
**10**	35.8	37.2	36.0	37.1	37.0	35.8
**11**	21.3	21.3	21.4	20.9	21.0	21.2
**12**	40.1	40.1	39.3	35.1	39.7	40.0
**13**	40.8	40.6	46.6	46.3	40.5	41.1
**14**	56.4	56.8	55.4	56.4	56.5	56.3
**15**	32.1	32.3	35.4	32.3	32.2	32.1
**16**	81.1	81.2	144.7	144.6	81.3	81.3
**17**	63.0	63.2	155.5	155.2	64.2	64.3
**18**	16.5	16.4	16.3	15.9	19.3	16.5
**19**	12.3	19.5	12.4	19.2	19.3	12.3
**20**	42.0	42.1	196.3	196.2	40.7	40.5
**21**	14.9	15.0	30.5	27.1	16.3	16.3
**22**	109.2	109.3			112.6	112.6
**23**	31.8	32.0			30.8	30.0
**24**	29.2	29.4			28.4	28.2
**25**	30.6	30.7			34.2	34.2
**26**	66.9	67.0			75.2	75.2
**27**	17.2	17.3			17.2	17.1
**22**-OCH_3_					47.2	47.2
Gal-(1 → 3)-skeleton						
**1'**	102.5	103.0		100.3	102.7	102.4
**2'**	73.1	73.5		73.2	73.3	73.3
**3'**	75.5	75.4		75.6	75.6	75.6
**4'**	79.8	79.8		81.0	81.0	81.0
**5'**	75.3	75.9		75.1	75.2	75.2
**6'**	60.6	61.0		60.4	60.4	60.5
Glc'-(1 → 4)-Gal						
**1''**	104.9	107.0		105.2	105.0	105.0
**2''**	81.2	75.2		86.1	86.1	86.1
**3''**	87.0	78.4		78.5	78.5	78.4
**4''**	70.4	72.4		71.8	71.8	71.8
**5''**	77.6	78.7		78.2	78.2	78.9
**6''**	63.0	63.1		63.2	63.2	63.2
Glc''-(1 → 2)-Glc'						
**1'''**	104.7			106.9	106.9	106.9
**2'''**	76.1			76.6	76.7	76.7
**3'''**	77.5			77.6	77.6	77.6
**4'''**	71.1			70.3	70.3	70.3
**5'''**	77.8			78.9	78.9	78.9
**6'''**	62.5			61.6	61.6	61.6
Xyl-(1 → 3)-Glc'						
**1'''**	104.9					
**2'''**	75.0					
**3'''**	78.5					
**4'''**	70.7					
**5'''**	67.2					
26-Glc						
**1''''**					105.2	105.2
**2''''**					75.2	75.2
**3''''**					78.6	78.6
**4''''**					71.7	71.7
**5''''**					78.5	78.5
**6''''**					62.9	62.9

**Fig. 6:**
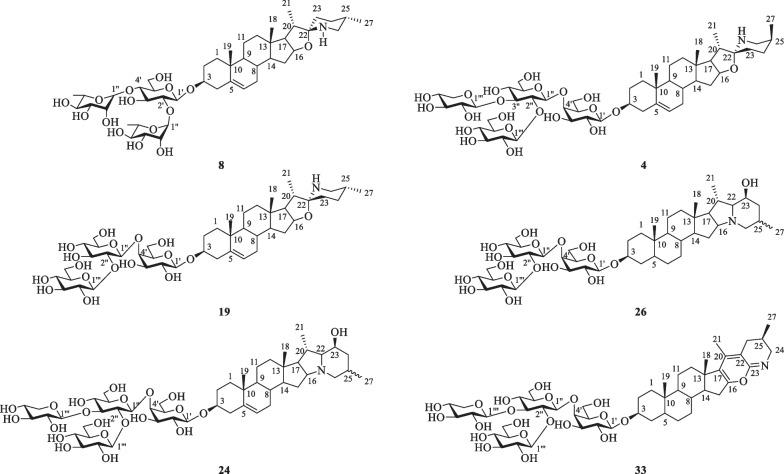
Representative six common steroidal alkaloids

In the ^13^C NMR spectra, spirosolane-type of steroidal alkaloids, characterizing a A/B/C/D ring system of C_27_ steroid scaffold (Table 3 and Fig. 6), show twenty-seven carbon signals generally containing four methyl carbon signals around at *δ*_C_ 16.0 (C-18), 19.0 (C-19), 15.0 (C-21), and 19.0 (C-27). Steroidal alkaloid can be simply recognized to be a steroidal saponin with a replacement of the oxygen atom in F ring by a nitrogen atom, resulting in the high-field shifting of the carbon chemical shifts of C-22 and C-26, from *δ*_C_ 109.0 and 67.0 to 98.0 and 47.5, respectively [30, 39, 40, 46]. In the ^13^C NMR spectra of the solanidane-type steroidal alkaloids, there were four methyl carbon signals around at *δ*_C_ 16.0 (C-18), 12.0 (C-19), 19.0 (C-21), and 22.0 (C-27) [30, 38, 39, 46] besides those characteristic carbon signals around at *δ*_C_ 69.0 (C-16), 78.0 (C-22) and 58.0 (C-26) [35, 41]. And, chemical shifts of the glycosyl anomeric carbon are at *δ*_C_ 100.0–108.0, especially when the glycosidation taking place at OH-3 of the steroidal alkaloids [47–49].

### Steroids and steroidal saponins

#### Steroids

**Fig. 7:**
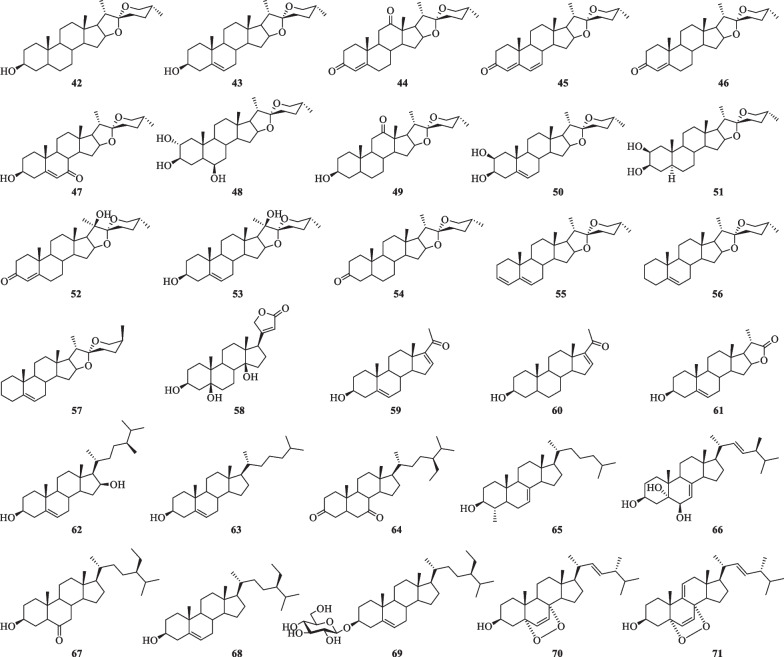
Chemical structures of steroids from *S. lyratum*

Cholestanol, containing a perhydrocyclopentenophenanthrene moiety (rings A, B, C and D) with a acyclic side-chain, has been considered as the precursor of furostanol and spirostanol (Fig. 7). At present, thirty steroids have been isolated from *S. lyratum* (**42**–**71**) (Table 4) [35, 36, 39, 47, 48, 50, 52].

#### Steroidal saponins

**Fig. 8:**
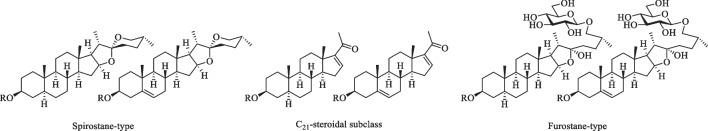
Sapogenin scaffolds of saponins reported in *S. lyratum*

**Fig. 9:**
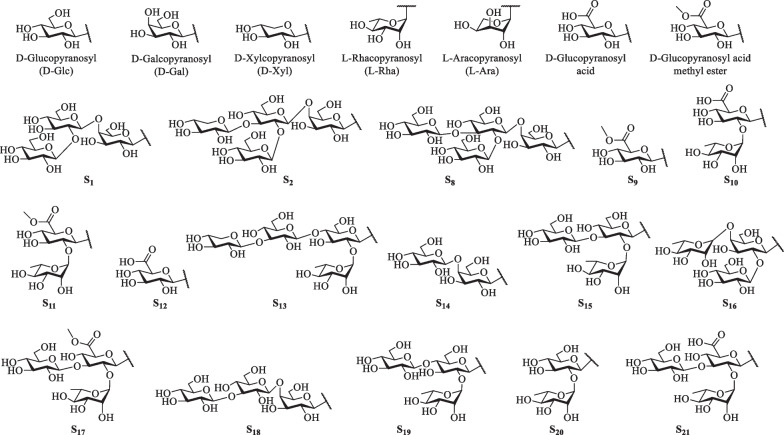
Glycones of saponins reported in *S. lyratum*

Steroidal saponins reported in *S. lyratum* are well-known characterized by possessing the steroid-derived aglycones normally consisting of a hydrophobic C_27_-skeleton of cholestane with an oxygen fused into the F ring, exemplified by the spirostanol (27 carbon atoms), furostanol (27 carbon atoms) and cholestanol (27 carbon atoms) as the most common scaffolds. Nevertheless, C_21_- steroidal saponins have also been reported to be existed in *S. lyratum* (Fig. 8). As is known, the remaining hydrophilic glycone unit of a steroidal saponin has been frequently reported to be substituted at the C-3 position of the sapogenin. D-Glc, D-Gal, D-Xyl, L-Rha, and L-arabinose (L-Ara) are the common members of the glycones, in which one to five monosaccharides linked linearly or with one or more branched chains, as shown in Fig. 9.

**Fig. 10:**
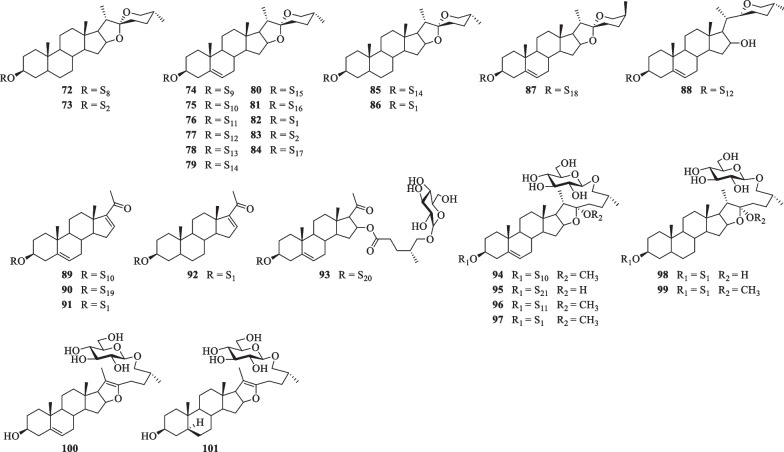
Chemical structures of steroidal saponins from *S. lyratum*

Till now, a total of thirty steroidal saponins (**72**–**101**) have been identified from *S. lyratum* (Table 5 and Fig. 10). Most of the isolated steroidal saponins of *S. lyratum* belong to the spirostane-type (**72**–**87**) [4, 22, 46, 47, 49], C_21_-steroidal subclass (**89**–**93**) [40, 46, 51] and furostanol-type (**94**–**101**) [22, 23, 40, 46]. Notably, when F-ring of a spirostanol is ring-opened, a new sapogenin skeleton of a furostanol is then afforded. As far as we know, the furostanol and its derivatives are the only reported ring-opened steroidal saponins ever isolated from *S. lyratum* up to now (**94**–**101**) [22, 23, 40, 46].

### NMR characteristics of steroidal saponins

**Fig. 11:**
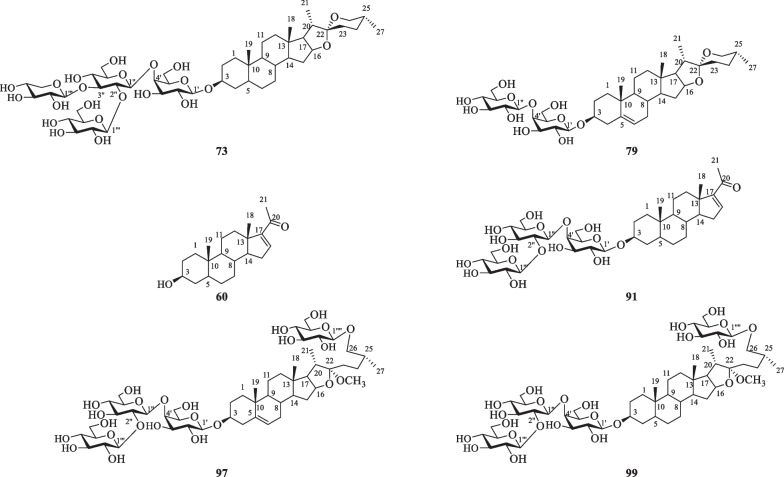
Representative six common steroidal saponins

In short, in the ^13^C NMR spectra, spirostanol and furostanol-types of steroidal saponins, characterizing a A/B/C/D ring system of C_27_-steroidal scaffold, show twenty-seven carbon signals generally containing four methyl carbon signals at *δ*_C_ 16.0 (C-18), 19.0 (C-19), 14.0 (C-21), and 17.0 (C-27) or two olefinic carbon signals at *δ*_C_ 140.0 (C-5) and 120.0 (C-6) [47, 49] (Table 6 and Fig. 11). The carbon chemical shift of CH_3_-19 will down-field shifted from *δ*_C_ 12.0 to *δ*_C_ 19.0 [47, 49], when the two methylenes at C-5,6 being dehydrogenated (-H_2_) to form a double bond [53]. The carbon resonances of C-16, 17, 22 (spiroketal carbon) and C-26 in a spirostanol-type steroidal saponin, were at about *δ*_C_ 81.0, 62.0, 109.0 and 67.0, respectively [47, 49], while those in a furostanol-type steroidal saponin were at about *δ*_C_ 81.0, 64.0, 112.0 and 75.0, respectively [22, 48]. It is worth noting that the proton chemical shift (*δ*_H_) difference (*∆*_ab_ = *δ*_a_-*δ*_b_) of the two geminal protons (Ha and Hb) of CH_2_-26 has been recognized for ascertaining 25*R* or 25*S* orientation of the CH_3_-27 [*∆*_ab_ ≤0.48 for 25*R*, *∆*_ab_≥ 0.57 for 25*S*] in the spirostanol and furostanol-types of steroidal saponins [45, 54, 55]. Similarly, the abovementioned empirical law for configuration assignment of C-25 applies equally well to spirosolane-type of steroidal alkaloids (Table 3).

### Terpenoids

**Fig. 12:**
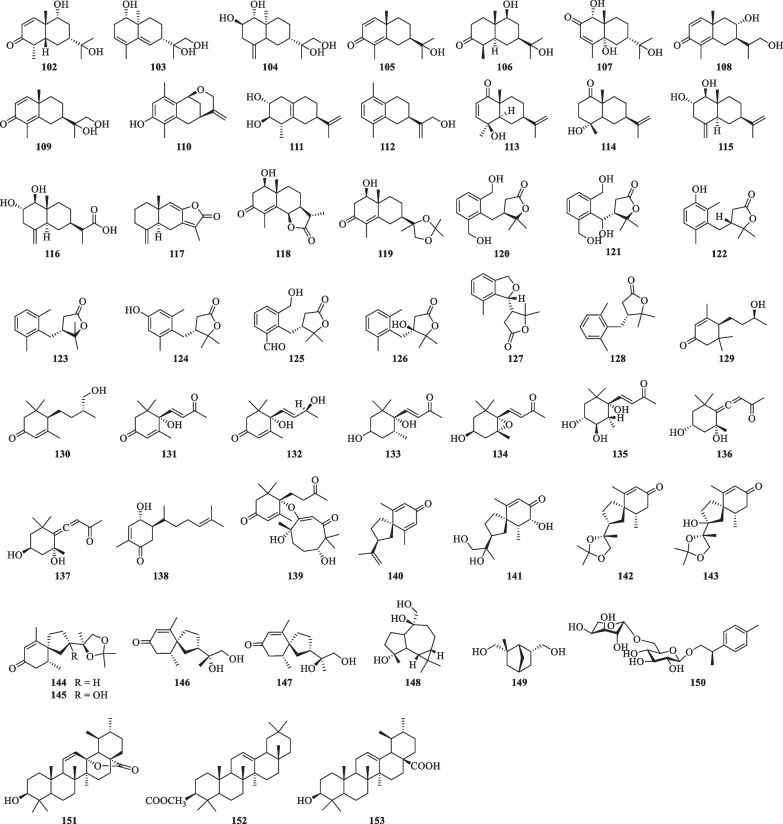
Chemical structures of terpenoids from *S. lyratum*

So far, fifty-one terpenoids, including sesquiterpenoids, monoterpenoids and triterpenoids (Table 7 and Fig. 12), have been isolated from *S. lyratum*. Among them, sesquiterpenoids are the most common terpenoids in *S. lyratum*, including eudesmane-type sesquiterpenoids (**102**–**119**) [52, 56–64] and the related derivatives (**120**–**128**) [39, 47, 58, 60, 62], monocyclic sesquiterpenoids (**129**–**139**) [25, 47, 56, 58, 62], vetispirane-type sesquiterpenoids (**140**–**147**) [1, 56, 62, 64], and guaiane-type sesquiterpenoids (**148**) [47]. In addition to the abovementioned constituents, two monoterpenoids (**149**–**150**) and three pentacyclic triterpenoids (**151**–**153**) have also been found in *S. lyratum*.

**Table 7:** Terpenoids isolated from *S. lyratum*

No	Compounds	Chemical formula	Molecular Wt	Refs.
**102**	Lyratol A	C_15_H_24_O_3_	252.1725	[56]
**103**	Lyratol B	C_15_H_24_O_3_	252.1725	[57]
**104**	Lyratol C	C_15_H_26_O_4_	270.1831	[58]
**105**	Dehydrocarlssone	C_15_H_22_O_2_	234.162	[52]
**106**	Lyratol G	C_15_H_26_O_3_	254.1882	[59]
**107**	Solajiangxin A	C_15_H_24_O_4_	268.1675	[60]
**108**	Solajiangxin G	C_15_H_22_O_3_	250.1529	[61]
**109**	Solajiangxin F	C_15_H_22_O_3_	250.1529	[61]
**110**	Solanoid A	C_15_H_18_O_2_	230.1307	[62]
**111**	Rishitin	C_14_H_22_O_2_	222.162	[63]
**112**	Solanoid B	C_15_H_20_O	216.1514	[62]
**113**	Solanoid D	C_15_H_22_O_2_	234.162	[62]
**114**	(4*R*,5*R*,7*R*,10*R*)-4-Hydroxyleudesmane-2,11-dien-1-one	C_15_H_22_O_2_	234.162	[62]
**115**	Nardoeudesmol A	C_15_H_24_O_2_	236.1776	[62]
**116**	Solajiangxin D	C_15_H_24_O_4_	268.1675	[60]
**117**	Atrectylsnollde I	C_15_H_18_O_2_	230.1307	[52]
**118**	1*β*-Hydroxy-1,2-dihydro-*α*-santonin	C_15_H_20_O_4_	264.1362	[59]
**119**	Solajiangxin H	C_18_H_28_O_4_	308.1988	[64]
**120**	Septemlobin G	C_15_H_20_O_4_	264.1362	[47]
**121**	Septemlobin H	C_15_H_20_O_5_	280.1311	[47]
**122**	Lycifuranone A	C_15_H_20_O_3_	248.1412	[62]
**123**	( +)-(*R*)-5,5-Dimethyl-4-(2,6-dimethylbenzyl)-solafuranone	C_15_H_20_O_2_	232.1463	[47]
**124**	Lyratol D	C_15_H_20_O_3_	248.1412	[58]
**125**	Solajiangxin B	C_15_H_18_O_4_	262.1205	[60]
**126**	Solanoid C	C_15_H_20_O_3_	248.1412	[62]
**127**	Solajiangxin C	C_15_H_18_O_3_	246.1256	[60]
**128**	Solafuranone	C_15_H_20_O_2_	232.1463	[39]
**129**	Blumenol C	C_13_H_22_O_2_	210.162	[47]
**130**	Blumenol	C_14_H_24_O_2_	224.1776	[47]
**131**	Dehydrovomifoliol	C_13_H_18_O_3_	222.1256	[58]
**132**	Blumenol A	C_13_H_20_O_3_	224.2412	[58]
**133**	Boscialin	C_13_H_22_O_3_	226.1529	[56]
**134**	3*β*-Hydroxyl-5*α*,6*α* -epoxy-7-megastigmen-9-one	C_13_H_20_O_3_	224.1412	[56]
**135**	Lyratol E	C_13_H_22_O_4_	242.1518	[56]
**136**	(4*R*)-4-(3-Oxo-1-butene-1-ylidene)-3*α*,5,5-trimethylcyclohexane-1*α*,3*β*-diol	C_13_H_20_O_3_	224.1412	[56]
**137**	Lyratol F	C_13_H_20_O_3_	224.1412	[56]
**138**	1*α* -Hydroxybisabol-2,10-dien-4-one	C_15_H_24_O_2_	236.1776	[62]
**139**	Solalyratin B	C_24_H_34_O_6_	418.2355	[25]
**140**	Anhydro-*β*-rotunol	C_15_H_20_O	216.1514	[62]
**141**	2-(1′,2′-Dihydroxyl-1'-methylethyl)-6,10-dimethyl-9-hydroxyspirodec-6-en-8-one	C_15_H_24_O_4_	268.1675	[56]
**142**	Solajiangxin E	C_18_H_28_O_3_	292.2038	[1]
**143**	2-Hydroxysolajiangxin E	C_18_H_28_O_4_	308.1988	[1]
**144**	Solajiangxin I	C_18_H_28_O_3_	292.2038	[64]
**145**	7-Hydroxysolajiangxin I	C_18_H_28_O_4_	308.1988	[64]
**146**	(1'*S*,2*R*,5*S*,10*R*)-2-(1',2'-Dihydroxy-l'-methylethyl)-6,l0-dimethylspiro[4,5]dec-6-en-8-one	C_15_H_24_O_3_	252.1725	[56]
**147**	(1'*R*,2*R*,5*S*,10*R*)-2-(1',2'-Dihydroxy-l'-methylethyl)-6,l0-dimethylspiro[4,5]dec-6-en-8-one	C_15_H_24_O_3_	252.1725	[56]
**148**	Pipelol A	C_15_H_26_O_3_	254.1882	[47]
**149**	Paeoveitol C	C_10_H_18_O_2_	170.1307	[47]
**150**	2-Phenylethyl-(6-*O*-*α*-L-arabinofuranosyl)-*O*-*β*-D-glucopyranoside	C_21_H_32_O_10_	444.1995	[47]
**151**	3-Hydroxyl-ll-ursen-28,13-olide	C_30_H_46_O_3_	454.3447	[47]
**152**	*β*-Amyrin acetate	C_32_H_52_O_2_	468.3967	[47]
**153**	Ursolic acid	C_30_H_48_O_3_	456.3603	[47]

### Nitrogenous compounds

**Fig. 13:**
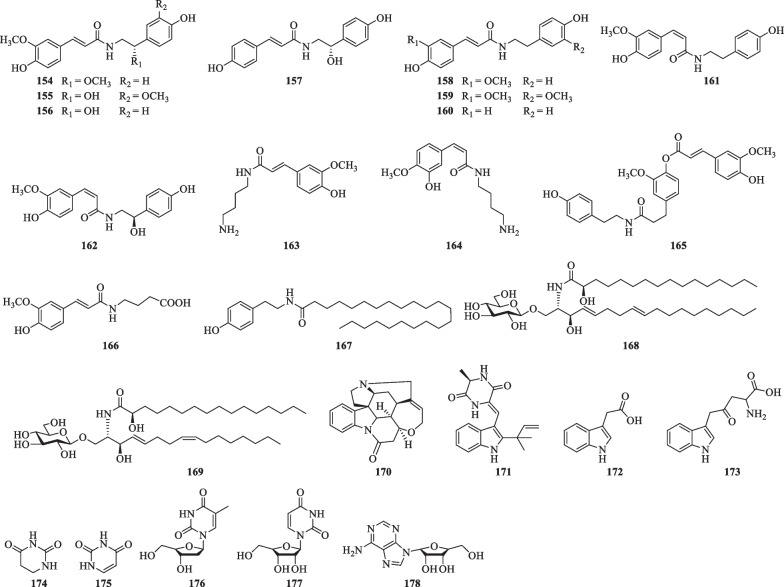
Chemical structures of nitrogenous compounds from *S. lyratum*

Nitrogenous compounds found in *S. lyratum* include arylamides (**154**–**167**) [36, 47, 65–68], aliphatic amides (**168**–**169**) [47], and other nitrogenous compounds (**170**–**178**) (Table 8 and Fig. 13) [47, 65].

**Table 8:** Nitrogenous compounds isolated from *S. lyratum*

No	Compounds	Chemical formula	Molecular Wt	Refs.
**154**	3-(4-Hydroxy-3-methoxyphenyl)-*N*-[2-(4-hydroxyphenyl)-2-methoxyethyl] acrylamide	C_19_H_21_NO_5_	343.142	[65]
**155**	*N*-*trans*-Feruloyl-3-methoxyoctopamine	C_19_H_21_NO_6_	359.1369	[36]
**156**	*N*-*trans*-Feruloyloctopamine	C_18_H_19_NO_5_	329.1263	[65]
**157**	(*E*)-*N*-(2-Hydroxy-2-(4-hydroxyphenyl)-ethyl)-3-(4-hdroxyphenyl) acrylamide	C_17_H_17_NO_4_	299.1158	[66]
**158**	*N*-*trans*-Feruloyltyramine	C_18_H_19_NO_4_	313.353	[65]
**159**	*N*-*trans*-Feruloyl-3-*O*-methyldopamine	C_19_H_21_NO_5_	343.142	[47, 65]
**160**	*N*-*trans*-Coumaroyltyramine	C_17_H_17_NO_3_	283.1208	[47, 67]
**161**	*N*-*cis*-Feruloyltyramine	C_18_H_19_NO_4_	313.353	[47]
**162**	*N*-*cis*-Femloyloctopamine	C_18_H_19_NO_5_	329.1263	[36]
**163**	(*E*)*-N*-(4-Aminobutyl)-3-(4-hydroxy-3-methoxyphenyl)acrylamide	C_14_H_20_N_2_O_3_	264.1474	[68]
**164**	(*Z*)*-N*-(4-Aminobutyl)-3-(4-hydroxy-3-methoxyphenyl)acrylamide	C_14_H_20_N_2_O_3_	264.1474	[68]
**165**	Hibiscuwanin B	C_28_H_29_NO_7_	491.1944	[47]
**166**	*N*-*trans*-Femloylbutyric acid	C_14_H_17_NO_5_	279.1107	[36]
**167**	*N*-Docosanoyltyrumine	C_30_H_53_NO_2_	459.4076	[47]
**168**	Soyacerebroside I	C_40_H_75_NO_9_	713.5442	[47]
**169**	Soyacerebroside II	C_40_H_75_NO_9_	713.5442	[47]
**170**	Strychnine	C_21_H_22_N_2_O_2_	334.1681	[65]
**171**	Neoechinulin A	C_19_H_21_N_2_O_3_	323.1634	[47]
**172**	*β*-Hydroxyindole acetic acid	C_10_H_9_NO_2_	175.0633	[47]
**173**	(*R*)-2-Amino-5-(1*H*-indol-3-yl)-4-oxopentanoic acid	C_13_H_14_N_2_O_3_	246.1004	[47]
**174**	Dihydrouracil	C_4_H_6_N_2_O_2_	114.0429	[47]
**175**	Uracil	C_4_H_4_N_2_O_2_	112.0273	[47]
**176**	Thymidine	C_10_H_14_N_2_O_5_	242.0903	[47]
**177**	Uridine	C_9_H_12_N_2_O_6_	244.0695	[47]
**178**	Adenosine	C_10_H_13_N_5_O_4_	267.0968	[47]

### Phenylpropanoids

#### Lignans

**Fig. 14:**
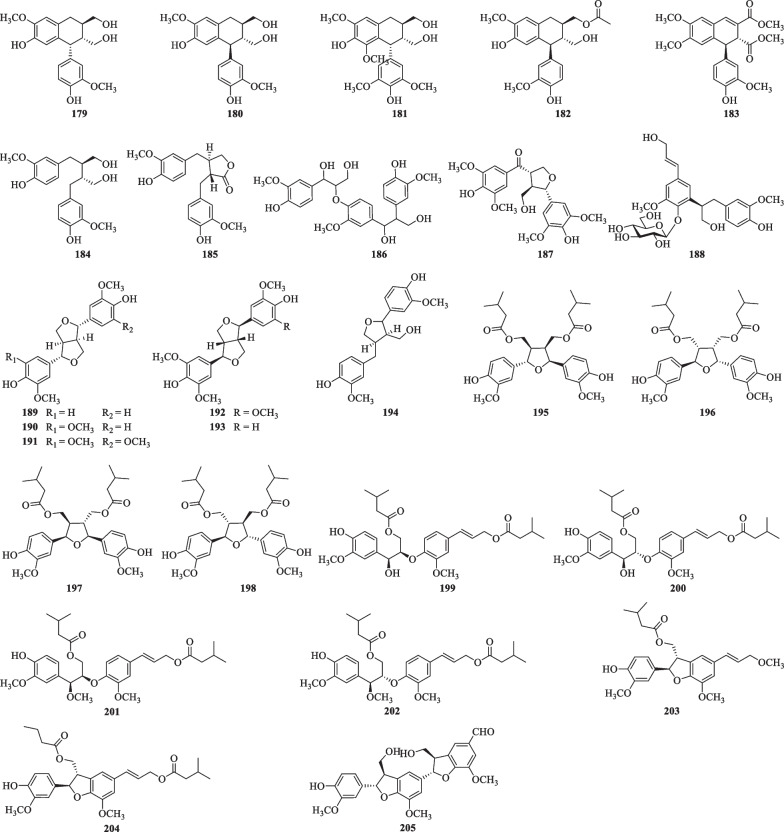
Chemical structures of lignans from *S. lyratum*

Till now, a total of twenty-eight lignans (**179**–**205**) have been isolated from *S. lyratum* (Table 9, Fig. 14), including simple lignans (**184**, **188**) [32, 47, 68], lignanolides (**185**) [47], cyclolignans (**179**–**183**) [32, 47], monoepoxyligans (**187**), bisepoxylignans (**189**–**193**) [32, 47, 66], and norlignans (**203**–**205**) [20, 32, 47]. Lignans with one or more isovaleroyloxyl substitution, as exemplified by compounds **195**–**204** [20], has been frequently uncovered from *S. lyratum* in recent years. Notably, neolignans including compounds **186**–**187** [47], and **195**–**202**, exhibited neuroprotective effects against human neuroblastoma SH-SY5Y cell injury induced by H_2_O_2_ [20].

**Table 9:** Lignans isolated from *S. lyratum*

No	Compounds	Chemical formula	Molecular Wt	Refs.
**179**	( +)-Isolariciresinol	C_22_H_22_O_8_	414.1315	[47]
**180**	*ent*-Isolariciresinol	C_22_H_22_O_8_	414.1315	[32, 47]
**181**	( +)-Lyoniresinol	C_22_H_28_O_8_	420.1784	[47]
**182**	Isolariciresinol-9-acetate	C_22_H_26_O_7_	402.1679	[66]
**183**	Aviculin	C_22_H_22_O_8_	414.1315	[32, 47]
**184**	(−)-Secoisolariciresinol	C_20_H_26_O_6_	362.1729	[32, 47]
**185**	( +)-Matairesinol	C_20_H_22_O_6_	358.1416	[47]
**186**	Leptolepisol D	C_27_H_32_O_10_	516.1995	[32, 47]
**187**	Ciwujiatone	C_22_H_26_O_9_	434.1577	[32, 47]
**188**	3-Methoxy-4-hydroxy-5-[(8'*S*)-3'-methoxy-4'-hydroxyphenylpropyl alcohol]-*E*-cinnamic alcohol-4-*O*-*β*-D-glucopyranoside	C_26_H_34_O_11_	522.2101	[68]
**189**	( +)-Pinoresinol	C_20_H_22_O_6_	358.1416	[32, 47]
**190**	( +)-Medioresinol	C_21_H_24_O_7_	388.1522	[47]
**191**	( +)-Syringaresinol	C_22_H_26_O_8_	418.1628	[32]
**192**	(−)-Syringaresinol	C_22_H_26_O_8_	418.1628	[47]
**193**	(−)-Epipinoresinol	C_21_H_24_O_7_	388.1522	[32, 47]
**194**	( +)-Lariciresinol	C_20_H_24_O_6_	360.1573	[47]
**195**	(7S,8R,7'R,8'R)-Solanumin A	C_30_H_40_O_9_	544.2672	[20]
**196**	(7R,8S,7'S,8'S)-Solanumin A	C_30_H_40_O_9_	544.2672	[20]
**197**	Solanumin B	C_30_H_40_O_9_	544.2672	[20]
**198**	Solanumin C	C_30_H_40_O_9_	544.2672	[20]
**199**	Solanumin D	C_30_H_40_O_9_	544.2672	[20]
**200**	Solanumin E	C_30_H_40_O_9_	544.2672	[20]
**201**	Solanumin F	C_31_H_42_O_9_	558.2829	[20]
**202**	Solanumin G	C_31_H_42_O_9_	558.2829	[20]
**203**	Solanumin H	C_26_H_32_O_7_	456.2148	[20]
**204**	Solanumin I	C_30_H_38_O_8_	526.2567	[20]
**205**	Cinncassin D	C_28_H_28_O_9_	508.1733	[47]

#### Coumarins, simple phenylpropanoids and their derivatives

**Fig. 15:**
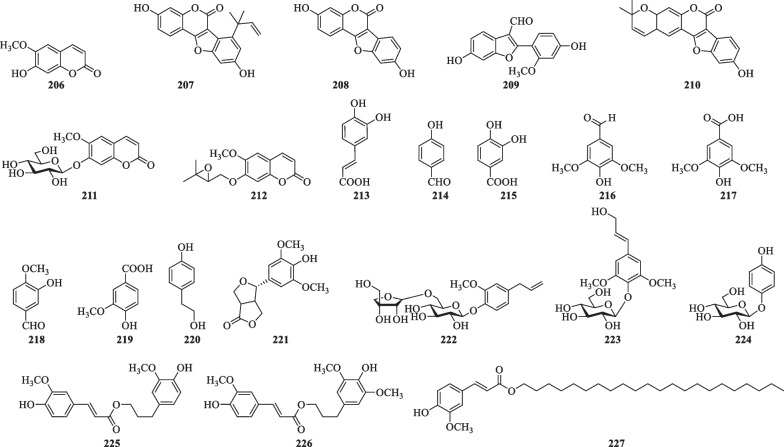
Chemical structures of coumarins, simple phenylpropanoids and their derivatives from *S. lyratum*

So far, seven coumarins (**206**–**212**) and eight simple phenylpropanoids (**213**–**220**) and their derivatives (**221**–**227**) have been isolated from *S. lyratum* as shown in Table 10 and Fig. 15.

**Table 10:** Coumarins, simple phenylpropanoids and their derivatives isolated from *S. lyratum*

No	Compounds	Chemical formula	Molecular Wt	Refs.
**206**	Scopoletin	C_10_H_8_O_4_	192.0423	[47]
**207**	Solalyratin A	C_20_H_16_O_5_	336.0998	[25]
**208**	Coumestrol	C_15_H_8_O_5_	268.0372	[25]
**209**	Puerariafuran	C_16_H_12_O_5_	284.0685	[25]
**210**	9-Hydroxy-2',2-dimethylpyrano[5',6':2,3]-coumestan	C_20_H_16_O_5_	336.0998	[25]
**211**	Magnolioside	C_16_H_18_O_9_	354.0951	[69]
**212**	7-(2,3-Epoxy-3-methyl-3-butyloxy)-6-methoxycoumarin	C_15_H_16_O_5_	276.0998	[47]
**213**	Caffeic acid	C_9_H_8_O_4_	180.0423	[47]
**214**	*p*-Hydroxybenzaldehyde	C_7_H_6_O_2_	122.0368	[47]
**215**	Protocatechuic acid	C_7_H_6_O_4_	154.0266	[47]
**216**	Syringaldehyde	C_9_H_10_O_4_	182.0579	[47]
**217**	Syringate	C_9_H_10_O_5_	198.0528	[47]
**218**	Isovanillin	C_8_H_8_O_3_	152.0473	[47]
**219**	Vanillic acid	C_8_H_8_O_4_	168.0423	[36]
**220**	*p*-Hydroxyphenethyl alcohol	C_8_H_10_O_2_	138.0681	[36]
**221**	Zhebeiresinol	C_14_H_16_O_6_	280.0947	[32, 47]
**222**	Eugenyl-*O*-*β-D*-apiofuranosyl-(1'' → 6')-*O*-*β-D*-glucopyranoside	C_20_H_28_O_11_	444.1632	[47]
**223**	Syringin	C_17_H_24_O_9_	372.1420	[32]
**224**	Arbutin	C_12_H_16_O_7_	272.0896	[70]
**225**	Dihydroconiferyl ferulate	C_20_H_22_O_6_	358.1416	[36]
**226**	Dihydrosinapyl ferulate	C_21_H_24_O_7_	388.1522	[36]
**227**	Docosvl ferulate	C_32_H_54_O_4_	502.4022	[47]

### Flavonoids

**Fig. 16:**
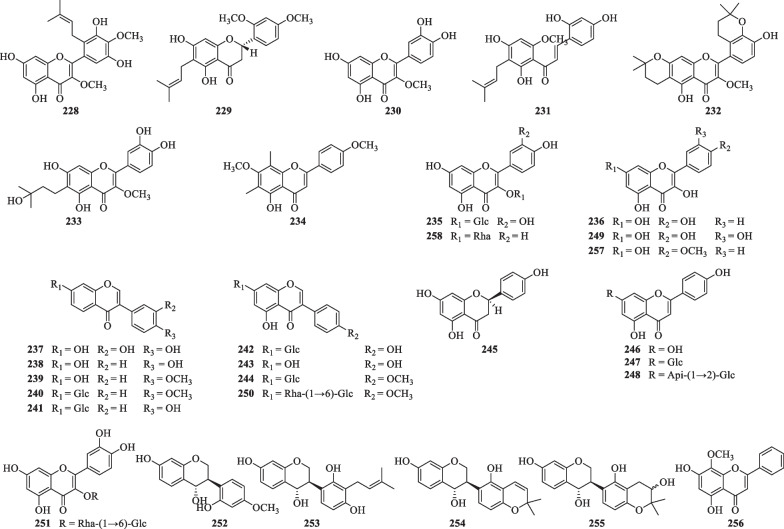
Chemical structures of flavonoids from *S. lyratum*

Thirty-one flavonoids (**228**–**258**) have been reported from *S. lyratum* (Table 11, Fig. 16), including flavonols (**228**, **230**, **232**–**236**, **246**–**249**, **251**, **256**–**258**) [47, 54, 68, 70–72], flavanones (**229**, **245**) [47, 72], isoflavones (**237**–**244**, **250**) [47, 54, 69, 71], chalcones (**231**) [47], and isoflavan-4-ols (**252**–**255**) [73], usually in the form of flavonoid glycoside with C-3 or C-7 substitution of the monosaccharides (D-Glc, D-Gal, D-Xyl, L-Rha, and L-Ara) and disaccharides [Rha (1 → 6) Glc, Xyl (1 → 2) Glc, Api (1 → 2) Glc and Xyl (1 → 6) Glc].

**Table 11:** Flavonoids isolated from *S. lyratum*

No	Compounds	Chemical formula	Molecular Wt	Refs.
**228**	5,7,3',5'-Tetrahydroxy-3,4'-dimethoxy-6'-prenylflavonoide	C_22_H_22_O_8_	414.1315	[47]
**229**	5,7-Dihydroxy-6-isopentenyl-2',4'-dimethoxydihydroflavone	C_22_H_24_O_6_	384.1573	[47]
**230**	3-Methoxyquercetin	C_16_H_12_O_7_	316.0583	[47]
**231**	7,9,2',4'-Tetrahydroxyl-8-isopentenyl-5-methoxychalcone	C_21_H_22_O_6_	370.1416	[47]
**232**	6,7-Bis-2',3'-(2,2-dimethyldihydropyrano)-5,4'-dihydroxy-3-methoxyflavone	C_26_H_28_O_7_	452.1835	[47]
**233**	Wightianin	C_21_H_22_O_8_	402.1315	[47]
**234**	5-Hydroxy-4',7-dimethoxy-6,8-dimethylflavone	C_19_H_18_O_5_	326.1154	[47]
**235**	Quercetin-3'-*O*-*β*-D-glucoside	C_21_H_20_O_12_	464.0955	[47]
**236**	Kaempferol	C_15_H_10_O_6_	286.0477	[47]
**237**	3'-Hydroxydaidzein	C_15_H_10_O_5_	270.0528	[47]
**238**	Daidzein	C_15_H_10_O_4_	254.0579	[47]
**239**	Formononetin	C_16_H_12_O_4_	268.0736	[71]
**240**	Ononin	C_22_H_22_O_9_	430.1264	[69]
**241**	Daidzin	C_21_H_20_O_9_	416.1107	[69]
**242**	Genistin	C_21_H_20_O_10_	432.1056	[69]
**243**	Genistein	C_15_H_10_O_5_	270.0528	[71]
**244**	5-Hydroxylononin	C_22_H_20_O_10_	446.1213	[69]
**245**	Naringenin	C_15_H_12_O_5_	272.0685	[72]
**246**	Apigenin	C_15_H_10_O_5_	270.0528	[71]
**247**	Apigenin-7-*O*-*β*-D-glycoside	C_21_H_20_O_10_	432.1056	[68]
**248**	Apigenin-7-*O*-*β*-D-apiofuranosyl(1 → 2)-*O*-*β*-D-glucopyranoside	C_26_H_28_O_14_	564.1479	[68]
**249**	Quercetin	C_15_H_10_O_7_	302.0427	[72]
**250**	Acacetin-7-*O*-rutinoside	C_28_H_32_O_15_	592.5174	[50]
**251**	Rutin	C_27_H_30_O_16_	610.1534	[67]
**252**	4,7,2'-Trihydroxy-4'-methoxyisoflavan	C_16_H_16_O_5_	288.0998	[73]
**253**	Lyratin A	C_20_H_22_O_5_	342.1467	[73]
**254**	Lyratin B	C_20_H_20_O_5_	340.1311	[73]
**255**	Lyratin C	C_20_H_22_O_6_	358.1416	[73]
**256**	Kaempferide	C_16_H_12_O_6_	300.0634	[70]
**257**	Wogonin	C_16_H_12_O_5_	284.0685	[47]
**258**	Afzelin	C_21_H_20_O_10_	432.1056	[70]

### Other compounds

**Fig. 17:**
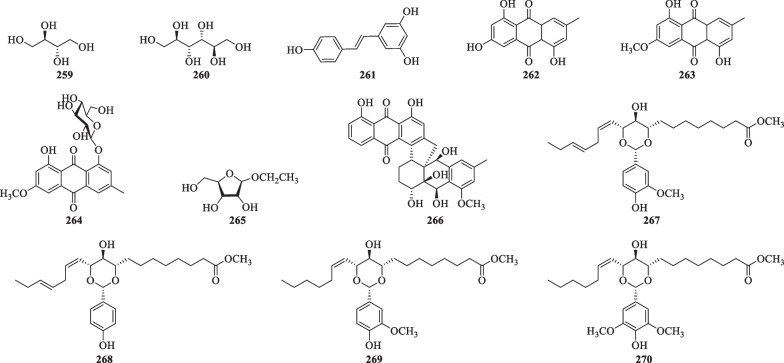
Chemical structures of other compounds reported from *S. lyratum*

In addition to the abovementioned constituents, other compounds including anthraquinones and fatty acids have also been isolated from *S. lyratum* (Table 12, Fig. 17).

**Table 12:** Other compounds isolated from *S. lyratum*

No	Compounds	Chemical formula	Molecular Wt	Refs.
**259**	Erythritol	C_4_H_10_O_4_	122.0579	[47]
**260**	Mannitol	C_6_H_14_O_6_	182.0790	[47]
**261**	3,4',5-Trihydroxystilbene	C_14_H_12_O_3_	228.0786	[72]
**262**	1,3,5-Trihydroxy-7-methylanthraquinone	C_15_H_12_O_5_	272.0685	[50]
**263**	1,5-Dihydroxy-3-methoxy-7-methylanthraquinone	C_16_H_14_O_5_	286.0841	[50]
**264**	Physcion-8-*O*-*β*-D-glucopyranoside	C_22_H_24_O_10_	446.1213	[50]
**265**	Ethyl-*α*-D-arabinofuranoside	C_7_H_14_O_5_	178.0841	[69]
**266**	Solanrubiellin A	C_31_H_28_O_9_	544.1733	[21]
**267**	Solacetal A	C_27_H_40_O_7_	476.2774	[26]
**268**	Solacetal B	C_26_H_38_O_6_	446.2628	[26]
**269**	Solacetal C	C_27_H_42_O_7_	478.2931	[26]
**270**	Solacetal D	C_28_H_44_O_8_	508.3036	[26]

## Pharmacology

Water decoction of the whole herb of *S. lyratum* was commonly used to treat various diseases, and the fresh whole herb was mashed to remedy herpes and warts for external use. Modern pharmacological evaluations revealed that extracts, fractions or compounds isolated from *S. lyratum* possessed various therapeutic potentials. Recently, the plant has been most extensively studied for its anti-cancer pharmacological properties. Meanwhile, other pharmacological effects such as anti-inflammatory, anti-oxidant, anti-microbial, anti-allergy, and hepatoprotective activities of *S. lyratum* have also been assessed, as summarized in Table 13.

**Table 13:** Pharmacological activities of *S. lyratum*

Bioactivities	Object	In vitro* / *in vivo	Mechanism	Extracts/Compounds	Refs.
Anti-lung cancer	mice with Lewis lung cancer	In vivo	Down-regulating the expression of Notch signaling pathway, improving the NK cell activity, increasing the number of CD_4_ cells, increasing sub-G_1_ peaks, and activating caspase-8, -9, and -3 protein, IC_50_ = 170 μg/mL	Total alkaloids, methanol and ethanol extracts	[19, 82, 98]
	Balb/C mouse with A549 lung cancer	In vivo*/*in vitro	Up-regulating the expression of bid mRNA, caspase-9, and inhibiting the tumor angiogenesis in Balb/C mice	50%, 80% ethanol extracts	[99]
	A549 cells and tumor-derived vascular endothelial cells	In vitro	Interfering with cell membrane lipid rafts, inhibiting tumor angiogenesis, inhibiting the activity of A549-derived exosomes, increasing immunity, suppressing Td-ECs migration, invasion, and tube formation, inhibiting pathways proteins, IC_50_ = 99.59–100 μg/mL	Compounds **1–4, 19**, **31**, **32**, **37–41**	[33, 43]
	A549 cells	In vitro	Increasing expression of I*κ*Ba and fas protein, decreasing expression of NF-*κ*B/p65, Survivin, fasL and p-I*κ*Ba proteins, arresting the cell cycle at the G_2_ phase, down-regulating the protein levels of PI3K, protein kinase B (Akt), Ras, microtubule-associated protein2 (MAP2), and VEGF, activating caspase-8 and caspase-3 proteins, IC_50_ = 6.54–13.49 μg/mL	Total alkaloids*,* ethanol and aqueous extracts, Compounds **42**, **44**, **55**, **231**	[47, 82, 86, 100–102]
	SPC-A-1 cells	In vitro	Inhibiting cell proliferation, promoted cell apoptosis, decreasing the expression of Bcl-xl, increasing the expression of fas, caspase-3, and bid, IC_50_ = 5–12.5 mg/mL	Ethanol and aqueous extracts	[103, 104]
Anti-hepatoma	Hep3B cells	In vitro	Inducing apoptosis and inhibit proliferation, **140** IC_50_ = 47.81 μM, **269** IC_50_ = 46.07 μM, **271** IC_50_ = 45.39 μM	Compounds **138**, **267**, **269**	[20, 26, 62]
	BEL-7402 cells	In vitro	Inducing apoptosis, activity similar to adriamycin and greater than 5-fluorouracil, IC_50_ = 0.39–23.0 μM	Compounds **36**, **72**, **73**, **81–83**, **86**	[22]
	Huh-7 cells	In vitro	Inducing apoptosis, activating p38 and Caspase-3 protein, IC_50_ = 15 mg/mL	Total alkaloids	[105]
	SMMC-7721 cells	In vitro	Up-regulating Fas, caspase-8, caspase-3, and p53, down-regulating FasL, survivin and Bcl-2 in the mitochondrial pathway, IC_50_ = 5 mg/L	75% ethanol extracts	[79]
	HepG2 cells	In vitro	Arresting the cell cycle at s-phase, inducing apoptosis, solamargine IC_50_ = 10.8 ± 0.1 μM, solasodine IC_50_ = 19.4 ± 0.4 μM, and solasonine IC_50_ = 91.8 ± 9.4 μM	Compounds **5**, **8**, **21**	[83]
Anti-sarcoma	S180 tumor-bearing mice	In vivo	Arresting the cell cycle at G_0_/G_1_ phase, improving immune response, promoting splenocytes proliferation, NK cell and Cytotoxic T lymphocyte (CTL) activity, interleukin-2 and interferon-*γ* production from splenocytes, and increasing the thymus and spleen indices to a certain extent	EtOAc fractions, total saponins, ethanol extracts	[77, 80, 106]
Anti-cervical cancer	HeLa cells	In vitro	Up-regulating the expression of caspase-3 mRNA, down-regulating the expression of survivin mRNA, activation of caspase-3, IC_50_ = 14.53 μg/mL	75% Ethanol and aqueous extracts*,* total saponins, compounds **44**, **55**, **59**, **74**, **75**, **177**, **229**, **231**, **237**	[42, 47, 107–109]
Anti-ovarian cancer	A2780 cells	In vitro	Inducing cell cycle arrest, enhanced reactive oxygen species (ROS) accumulation, activating the p53 signaling pathway, increasing the percentage of Cluster of Differentiation 86 (CD86 +) cells, decreasing the percentage of Cluster of Differentiation 26 (CD26 +) cells, and down-regulating expression of Bcl-2 mRNA	Ethanol and aqueous extracts	[110]
	HO8910 cells	In vitro	Inducing apoptosis, inhibiting proliferation in a dose-dependent, IC_50_ = 5 μg/mL	75% Ethanol extracts	[111]
	SKOV3 cells	In vitro	Arresting the cell cycle at the G_1_/S phase, up-regulating the expression of caspase-3, caspase-9 mRNA anti-tumor effect, and increasing the lactate dehydrogenase (LDH) release, IC_50_ = 4.51–7.78 μg/mL	90% Ethanol extracts	[112]
Anti-breast cancer	CHO cells	In vitro	Arresting the cell cycle at G_2_ phase, and inhibiting proliferation of CHO cells, IC_50_ = 0.5–1 g/mL	Aqueous extracts	[113]
	MCF-7 cell	In vitro	Up-regulating the expression of Bax mRNA and down-regulating the expression of survivin mRNA, IC_50_ = 160 μg/mL	Total saponins	[114]
Anti-oral cancer	HSC-3, SAS, and CAL-27 cell	In vitro	Arresting the cell cycle at G0/G1 phase, suppressing the anti-apoptotic proteins Bcl-2 and Bcl-xl, increasing the pro-apoptotic proteins Bax and Bad, promoting the production of ROS and Ca^2+^, decreasing the mitochondrial membrane potential, stimulating NO production, and activating caspase-8, -9, and -3 proteins activities, IC_50_ = 40 μg/mL	Chloroform extracts	[115]
Anti-stomach cancer	BGC823 cells	In vitro	Blocking the cell cycle in the G_1_/M phase, inducing apoptosis and inhibiting proliferation, IC_50_ = 25 μg/mL	Compounds **44**, **55**, **231**	[47]
	SGC-7901 cells	In vitro	Down-regulating expression of Bcl-xl mRNA and proteins, up-regulating expression of bid mRNA and proteins, caspase-9 and bid genes, strengthening the activity of Caspase-3; blocking the cell cycle in the G_2_/M phase, IC_50_ = 12.45–47.65 g/L	Aqueous extracts, total saponin, compounds **8**, 8**6**	[3, 116, 117]
Anti-colon cancer	HT-29 cells	In vitro	Down-regulating expression of survivin gene, up-regulating the expression of Caspase-3, 8, 9 mRNA and proteins; down-regulating the expression of Notch l mRNA, influencing the Notch signaling pathway to inhibit colorectal cancer cell proliferation and inducing apoptosis	Aqueous extracts	[118]
	CT-26 cells	In vitro	Increasing caspase-independent apoptosis associated with increased nuclear translocation of AIF, IC_50_ = 3.5 μM	Compounds **10**, **105**	[74]
	HT-29 cells	In vitro	Inducing apoptosis and inhibiting proliferation, ED_50_ = 1.9–3.7 μg/mL	Compounds **107**, **116**, **125**, **127**, **134**, **136**, **142**, **143**	[1, 60, 119]
Anti-leukemia	Leukemia mice	In vivo	Inhibiting the precursors of T cells and B cells, promoting the precursors of macrophages, increasing macrophage and NK cell activities, promoting the activity of macrophage phagocytosis in the peripheral blood mononuclear cells (PBMC) and peritoneal cells	Ethanol extracts	[78]
	HL-60 cells	In vitro	Up-regulating the expression of Bax mRNA, down-regulating expression of Bcl-2 mRNA, increasing Bax/ Bcl-2 protein ratio, IC_50_ = 3.5 mg/mL	Aqueous extracts	[120]
	P-388 cells	In vitro	Inhibiting proliferation and inducing apoptosis, ED_50_ = 2.7–3.1 μg/mL	Compounds **134**, **136**	[119]
Anti-prostate cancer	DU-145 cells and xenograft athymic nude mice	In vitro*/*in vivo	Blocking the expression of cell cycle proteins (Cyclin D1, Cyclin E1, CDK2, CDK4, CDK6, and P21) and inducing apoptosis via ROS and activation of the P38 pathway, IC_50_ = 32.18 μM	Steroidal glycoalkaloid	[121]
Anti-bone cancer	U-2 OS cells	In vitro	Arresting the cell cycle at the G_1_ phase, promoting the production of ROS and NO, decreasing the levels of mitochondrial membrane potential and promoting the activations of caspase-8, 9, 3; promoting the Bax level and release of cytochrome C, IC_50_ = 25 μg/mL	50% Ethanol extracts	[122]
Anti-neuroblastoma	SH-SY5Y cells	In vitro	Increasing the expression of Bcl-2 protein, and inhibiting the expression of Bax protein in tert-Butyl hydroperoxide (tBHP)-induced SH-SY5Y cells. inhibiting tBHP-induced ROS production, IC_50_ = 25–50 μM	Total alkaloids, compounds **194**–**203**	[20, 123]
Anti-inflammatory	SD rats	In vivo	Decreasing the content of PGE2 and cyclooxygenase-2 (COX-2) in serum	Aqueous and ethanol extracts, total saponins	[85, 86, 124]
	polymorphonuclear leukocytes of rats	In vivo	Inhibiting the release of *β*-glucuronidase, IC_50_ = 10 μM	Compounds **252**–**255**	[73]
Anti-microbial	*Staphylococcus aureus, Escherichia coli, Salmonella, Candida albicans, Pseudomonas aeruginosa*	In vitro	Inhibiting the growth of *Staphylococcus aureus*, *Escherichia coli, salmonella,* and *Candida albicans*, MIC = 100 mg/mL; pseudomonas aeruginosa, MIC = 50 mg/mL	Crude extracts, polysaccharides	[90]
	*Gram-positive bacteria*	In vitro	Inhibiting the *S. aureus* and *E. faecalis*, MIC = 2–10 μM	Compound **266**	[21]
Anti-allergy	Normal mice	In vivo	Inhibiting the histamine release, adding the level of cAMP, inhibiting overexpression of L-histamine decarboxylase mRNA	Aqueous extracts	[2]
	Mast cells	In vitro	Reducing the expression level of the mRNA of histidine decarboxylase (HDC), affecting IgE-mediated anaphylactic reaction and substance P-induced HDC mRNA over-expression	Aqueous extracts	[93]
	Normal mice	In vivo	Inhibiting the allergy to peritoneal mast cell histamine, delaying the kinetics of Low-Density Lipoprotein (LDL) oxidation, increasing the activity of peroxidase (POD) and superoxide dismutase (SOD), reducing the activity of malonaldehyde (MDA)	Aqueous extracts	[88]
Antioxidant activity	SH-SYSY cells	In vitro	Preserving mitochondrial membrane potential and reducing oxidative stress, LC_50_ = 4.64 μM, 457.12 μM, respectively; inhibiting tBHP-induced ROS production, oxidative stress, IC_50_ = 20 mg/L	Compounds **9**,**10** Total alkaloids	[87, 123]
	DPPH	In vitro	Scavenging activity of the stable DPPH free radical, IC_50_ = 5.98–23.16 mg/L	Ethyl acetate extracts, compounds **236, 248, 249, 251, 258**	[70]
	DPPH	In vitro	Scavenging free radical, ethanol extract IC_50_ = 0.23 mg/mL, ethyl acetate extract IC_50_ = 1.01 mg/mL, **251** IC_50_ = 3.30 mg/mL**, 253** IC_50_ = 6.73 mg/mL	Ethanol and ethyl acetate extract of *S. lyratum* fruits, compounds **249, 251**	[89]
Hepatoprotective activity	CCl4 induce mice	In vivo	Decreasing alanine aminotransferase (ALT), aspartate aminotransferase (AST), and alkaline phosphatase (ALP) contents, reducing CCl_4_-induced liver injury, anti-lipid peroxidation effect, decreasing transaminase activities in serum	Ethanol extracts	[91, 92]
Molluscicidal activity	Snails	In vitro	Having a molluscicidal effect, IC_50_ = 30–50 mg/mL	Ethanol extracts, compound **21**	[95]

### Anti-cancer

#### Extracts and fractions

The heat-clearing and detoxicating property of *S. lyratum* is favorable in the treatment of cancer [17, 19, 21]. It has been reported that *S. lyratum* treats various cancers by inhibiting the tumor growth [74, 75], enhancing immunity [76, 78] and inducing apoptosis via activating both extrinsic and intrinsic apoptotic pathways [27, 34, 79].

In S_180_ tumor-bearing mice, both ethanol and aqueous extracts of *S. lyratum* could improve immune function and exhibited anti-cancer potential with certain tumor inhibitory effect by improving the activities of natural killer (NK) and cluster of differentiation 4 (CD4) cells, and elevating the contents of serum Interleukin-2 (IL-2) and tumor necrosis factor-*α* (TNF-*α*) [77, 78, 80]. Total alkaloids from *S. lyratum* (SLTA, 24 mL/kg) could inhibit the tumor growth in mice with Lewis lung cancer, and when combined with cisplatin, a synergistic effect had been shown to down-regulate the mRNA expression of Notch1, Notch3 and Jagged1 in Notch signaling pathway [81]. In addition, the hexane fraction of the methanol extract (50 mg/kg) of *S. lyratum* showed similar inhibitory activity on tumor growth in mice with Lewis lung carcinoma tumor, potentially acting through up-regulating Fas, caspase-8, caspase-3, and p53, and down-regulating FasL and B-cell lymphoma-2 (Bcl-2) in the mitochondrial pathway [27, 79].

Further studies revealed that the 70% ethanol extract of *S. lyratum* (SLE) could suppress tumor angiogenesis in vitro by repressing migration, invasion, and tube formation of tumor-derived vascular endothelial cells (Td-ECs). The mechanism of the anti-angiogenic effect of SLE may be related to the inhibitory activity of vascular endothelial growth factor (VEGF) via reducing the number of lipid rafts in the cell membrane [43] and interfering with the lipid rafts by agglutinating cell membrane cholesterol [75]. These changes led to the inhibition of VEGFR2 phosphorylation and activation of its downstream signaling molecules, thereby inhibiting tumor angiogenesis [43]. In addition, SLTA could induce apoptosis of lung carcinoma A549 cells by inhibiting the nuclear factor-kappa B (NF-*κ*B) signaling pathway [82], while glycoalkaloids of *S. lyratum* (SLGS) significantly inhibited the activity of A549-derived exosomes with IC_50_ = 99.59 μg/mL [43].

#### Compounds

The cytotoxic tests involved in most in vitro studies of *S. lyratum* have shown that the compounds isolated from *S. lyratum* possess a good cytotoxic potential for several cancer cells.

In the process of cytotoxic investigation by MTT assay and flow cytometry, the characteristic ompounds **5**, **8**, **21** from the methanolic extract of *S. lyratum* showed significant cytotoxicities against huh-7 and HepG2 cell lines with IC_50_ values of 9.6 ± 0.5 and 10.8 ± 0.1 μM, 11.7 ± 0.3 and 19.4 ± 0.4 μM, and 10.3 ± 1.5 and 91.8 ± 9.4 μM, respectively. The mechanism was attributed to cell cycle arrest at S-phase [83]. while sesquiterpenoids **104**, **106**, **108**, **109**, **116**, **118**, **124**, **126**, **127**, **132**, **135**, **142**, and **143** were evaluated for their cytotoxicity activities with IC_50_ 1.9–8.6 μg/mL against HONE-1 cells [1, 57, 58, 60, 61]. Among them, compounds **126**–**127** showed potent cytotoxicity activity with IC_50_ 2.1 and 1.9 μg/mL, slightly weaker than the positive controls etoposide and cisplatin (IC_50_ 1.6 and 1.7 μg/mL) [1, 57, 58, 60, 61]. Notably, the IC_50_ differences of the positive controls (etoposide and cisplatin) may have been caused by the operation of the author, so the experimental cytotoxicity results need to be further verified.

Further, the cytotoxic potentials of nine steroids saponins and alkaloids (**36, 72**, **73**, **81–83, 86**) against ASGC7901 and BEL-7402 cancer cell lines were tested, and compounds **72**, **73**, and **83** showed attractive antiproliferative activities with respective IC_50_ values of 6.39–9.11 μM, 3.19–8.86 μM and 0.39–1.16 μM, as compared with IC_50_ values of 0.17–5.34 μM and 8.15–23.06 μM of positive control adriamycin and 5-fluorouracil, respectively [22]. Another, a glycoalkaloid (**10**) exhibited significant cytotoxicity against mouse colon cancer CT-26 cells with IC_50_ 3.5 μM, as compared to IC_50_ values of 1.8 μM of positive control etoposide, in clue of the inhibition on the expressions of survivin and NF-*κ*B/p65 and the induction of the AIF nuclear translocation [74]. Besides those characteristic constituents of *S. lyratum*, four other compounds **267–270** have been evaluated their cytotoxicities against hepatocellular carcinoma cell lines, and **267** and **269** showed significant inhibitory activities against HepG2 cell lines with IC_50_ values of 46.07 μM and 45.39 μM, respectively [26].

### Anti-inflammatory

#### Extracts and fractions

Inflammation is closely related to cancer disease [84]. The detoxication and detumescence effect of *S. lyratum* can be used as a supplement to modern anti-inflammatory agents.

Total alkaloid fraction from the 70% ethanol extract of *S. lyratum* significantly relieved the inflammatory effect of the lipopolysaccharide-stimulated RAW264.7 macrophages for 48 h. Further evaluation revealed that this total alkaloid fraction could inhibit the release of Cyclooxygenase-2 (COX-2), and Prostaglandin E2 (PGE2) from lipopolysaccharide-stimulated RAW264.7 macrophages [85].

#### Compounds

In vitro, diosgenin-3-*O*-*α*-L-rhamnosyl-(1 → 2)-*O-β*-D-glucopyranosiduronic acid (**75**) could inhibit the lipopolysaccharide-induced expression of intercellular cell adhesion molecule-1 (ICAM-1) protein at 16 μg/mL, and exhibited anti-inflammatory activities [86]. In addition, in the anti-inflammatory experiments with polymorphonuclear leukocytes of rats (rat PMNs) with ginkgolide B as the positive control, compounds **139**, **207**–**210** showed significant *β*-glucuronidase inhibitory activities with IC_50_ values range of 6.3–9.1 μM [25], while four 4-hydroxyisoflavans **252**–**255** afforded anti-inflammatory activities with inhibitory ratios release of *β*-glucuronidase in the range of 30.3–38.6% at 10 μM [73].

### Antioxidant activity

#### Extracts

Modern pharmacological studies have revealed that cancer or other diseases are primarily associated with the production and accumulation of excessive free radicals [87], which are commonly produced by the continuous contact between our body and the outside world. Thus, antioxidants can effectively relieve the harmful effects of free radicals. It has been confirmed that *S. lyratum* extracts and compounds possess significant antioxidant activities.

50% Ethanol extract of *S. lyratum* (10 μg/mL) could protect against oxidized low-density lipoprotein (Ox-LDL)-induced injury in cultured human umbilical vein endothelial cells (HUVECs) by direct antioxidative action [88]. In the DPPH radical-scavenging tests in vitro using the spectrophotometric method with vitamin C as the positive control, the ethanol and ethyl acetate extracts from *S. lyratum* showed antioxidative potential with IC_50_ of 0.230 mg/mL and 1.010 mg/mL, respectively [89].

#### Compounds

Five flavones **236**, **248**, **249**, **251**, and **258** from the ethanol extracts of *S. lyratum* possessed the capability of scavenging DPPH free radicals with IC_50_ values of 2.56–21.33 μg/mL [70]. It seems that the glycosidation of the flavone C-3 and C-5 is essential for the scavenging of DPPH free radicals.

### Antimicrobial

#### Extracts and fractions

There were reports verified the antibacterial potential of *S. lyratum* extracts. For example, the water-soluble polysaccharide of *S. lyratum* exerted a significant antibacterial activity against *Staphylococcus aureus*, *Salmonella*, *Pasteurella*, *Escherichia coli*, *Candida albicans,* and *Pseudomonas aeruginosa*. The inhibition zone diameters were > 13 mm at a concentration of 120 mg/mL [90].

#### Compounds

Several gram-positive bacteria (*S. aureus* and *Enterococcus faecalis*) were used to assess the antimicrobial activity of a new compound **266** from *S. lyratum*, with minimum inhibitory concentration (MIC) values of 2.0 μM (1.08 μg/mL) and 10.0 μM (5.44 μg/mL), respectively [21].

### Other activities

The extracts of *S. lyratum* showed a therapeutic potential on the tetrachloride-induced liver damage in rats, by decreasing significantly the activity of transaminase in rat serum [91, 92]. Further, there was an in vivo report revealed that the aqueous extract of *S. lyratum* possessed strong antiallergy activity [93] by inhibiting dose-dependently the histamine release from the rat peritoneal mast cells and decreasing the mRNA expression of L-histamine decarboxylase [94]. Lastly, the ethanol extract of *S. lyratum* possesses molluscicidal activities with IC_50_ values of 30–50 mg/mL [95].

Notably, several Chinese patent prescriptions with *S. lyratum* as the major component herb, showed significant anticancer efficacies in reported clinic trials. For example, 'Baiyingtang' (composed of *S. lyratum*, *Herba Patriniae*, *Houttuynia cordata*, *Lilium brownii* var., *Asparaguscochinchinensis*(Lour.)Merr.) retention enema could reduce plasma transforming growth factor-*β*1 (TGF-*β*1), interleukin-6 (IL-6) levels and increase plasma IL-4 levels in patients with pelvic tumors receiving radiotherapy [96]. 'Baiying decoction' (composed of *S. lyratum*, *Ophiocordyceps sinensis*, *Houttuynia cordata*, *Lilium brownii* var., *Asparaguscochinchinensis*(Lour.)Merr.) treatment could ameliorate the marrow suppression and the quality of life in patients with advance non-small cell lung cancer, with high safety [97].

## Toxicology

Up to now, the toxicity studies of the isolated compounds and extracts of *S. lyratum* may have been overlooked by researchers, while few studies have found the toxic potential of *Solanum* glycoalkaloid [125]. The toxic properties of Glycoalkaloids including solamargine (**8**) have been reviewed by Sinani AI S.S.S. et al. are due to (1) their ability to disrupt cell-membrane function by complexation with membrane 3*β*-hydroxysterols to form aggregates and damage the membrane integrity [126], (2) their anti-acetylcholinesterase activity on the central nervous system [126, 127], and (3) changes caused by them in active transport of ions through membranes, resulting in disorders in general body metabolism [126]. Additionally, in the acute toxicity experiment with rats, neither mortality nor clinical alterations were shown, except for the mild transient diarrhea with 70% ethanol extract of *S. lyratum* at 5000 mg/kg [31]. In future, more pharmacological evidences should be sought on the possible adverse effects and the toxicities of *S. lyratum* extracts and their bioactive constituents when used in treatments of acute, subchronic, or chronic diseases. Further, more clinical trials must be conducted to evaluate the safety and clinical efficacy of *S. lyratum* in humans.

## Conclusion

This review summarized the latest advancements of *S. lyratum* in botany, traditional uses, phytochemistry, pharmacology, and toxicology. Phytochemical and pharmacological studies have validated many modern usages of this plant. A total of 270 chemical constituents have been isolated from *S. lyratum*, including steroidal alkaloids, steroidal saponins, terpenoids, nitrogenous compounds, phenylpropanoids, flavonoids, etc. It has been popular in traditional practices due to its potential efficacy on cancer and inflammation, and showed important biological properties in scientific investigations. In the phytochemical analysis of *S. lyratum* extracts, aqueous and ethanol extracts were commonly acquired from *S. lyratum*, whose main components included total alkaloids and total saponins. In modern pharmacological studies, compounds and extracts from *S. lyratum* were evaluated in vivo and in vitro, and their anticancer and cytotoxic, anti-inflammatory, antioxidant, antimicrobial, anti-allergy, and hepatoprotective activities have been demonstrated. However, many aspects of *S. lyratum* have not been studied yet and some relative studies on *S. lyratum* should be further explored in the following aspects in the future.

Firstly, the pharmacological activities are mostly proven from the aqueous and ethanol extracts from *S. lyratum*, while insufficient pharmacological studies have been conducted on pure compounds. In addition, some activities are lacking comparisons to standards or positive and negative controls. Other studies especially on anticancer and anti-inflammatory activities have shown that the IC_50_ values of the test extracts/compounds of *S. lyratum* are above 200 μg/mL, which can be considered that such extracts/compounds are actually poorly active.

Secondly, pharmacological studies were mostly performed in cell models and animals while investigations in humans have been seldomly performed. Hence, the future investigation should be focused on the bioactivity of *S. lyratum* in various clinical studies with humans. In addition, the DPPH radical scavenging test and antimicrobial activities also should be guaranteed in vivo, instead of solely relying on method models in vitro.

Thirdly, global quality control standards of *S. lyratum* are needed urgently and should be improved. Simultaneous qualitative and quantitative measures are recommended to be used for those major active constituents of *S. lyratum*.

Finally, in toxicological studies of *S. lyratum*, no unequivocal proof of the toxicological activities in human exists. Further, relationship studies between systematic toxicity and safety evaluation are still needed to assure safety for clinical application in the future. Pharmacological effects of *S. lyratum* have been demonstrated by ethanol and aqueous extracts of high doses, the effectiveness of high doses extracts in treating diseases provides the possibility of finding active compounds. Therefore, it is important to study the therapeutic window (the range between the doses that produce the desired therapeutic effect and doses that produce toxicity) and the long-term in vivo toxicity for further research on *S. lyratum.*

In summary, *S. lyratum* can be considered as an important and valuable resources for human's health. Further research is needed in terms of quality control, toxicity and pharmacological mechanism to provide a theoretical basis for exploitation of the medicinal functions of *S. lyratum*.
